# Failure to breathe persists without air hunger or alarm following amygdala seizures

**DOI:** 10.1172/jci.insight.172423

**Published:** 2023-11-22

**Authors:** Gail I.S. Harmata, Ariane E. Rhone, Christopher K. Kovach, Sukhbinder Kumar, Md Rakibul Mowla, Rup K. Sainju, Yasunori Nagahama, Hiroyuki Oya, Brian K. Gehlbach, Michael A. Ciliberto, Rashmi N. Mueller, Hiroto Kawasaki, Kyle T.S. Pattinson, Kristina Simonyan, Paul W. Davenport, Matthew A. Howard, Mitchell Steinschneider, Aubrey C. Chan, George B. Richerson, John A. Wemmie, Brian J. Dlouhy

**Affiliations:** 1 Department of Neurosurgery,; 2 Iowa Neuroscience Institute,; 3 Pappajohn Biomedical Institute,; 4 Interdisciplinary Graduate Program in Neuroscience,; 5 Pharmacological Sciences Training Program,; 6 Department of Psychiatry,; 7 Department of Neurology,; 8 Department of Internal Medicine,; 9 Department of Pediatrics, and; 10 Department of Anesthesia, University of Iowa, Iowa City, Iowa, USA.; 11 Nuffield Department of Clinical Neurosciences, University of Oxford, John Radcliffe Hospital, Oxford, United Kingdom.; 12 Department of Otolaryngology–Head and Neck Surgery, Massachusetts Eye and Ear and Harvard Medical School, Boston, Massachusetts, USA.; 13 Department of Physiological Sciences, University of Florida, Gainesville, Florida, USA.; 14 Department of Neurology, Albert Einstein College of Medicine, Bronx, New York, USA.; 15 Department of Molecular Physiology and Biophysics, University of Iowa, Iowa City, Iowa, USA.; 16 Department of Veterans Affairs Medical Center, Iowa City, Iowa, USA.

**Keywords:** Neuroscience, Epilepsy, Respiration, Seizures

## Abstract

Postictal apnea is thought to be a major cause of sudden unexpected death in epilepsy (SUDEP). However, the mechanisms underlying postictal apnea are unknown. To understand causes of postictal apnea, we used a multimodal approach to study brain mechanisms of breathing control in 20 patients (ranging from pediatric to adult) undergoing intracranial electroencephalography for intractable epilepsy. Our results indicate that amygdala seizures can cause postictal apnea. Moreover, we identified a distinct region within the amygdala where electrical stimulation was sufficient to reproduce prolonged breathing loss persisting well beyond the end of stimulation. The persistent apnea was resistant to rising CO_2_ levels, and air hunger failed to occur, suggesting impaired CO_2_ chemosensitivity. Using es-fMRI, a potentially novel approach combining electrical stimulation with functional MRI, we found that amygdala stimulation altered blood oxygen level–dependent (BOLD) activity in the pons/medulla and ventral insula. Together, these findings suggest that seizure activity in a focal subregion of the amygdala is sufficient to suppress breathing and air hunger for prolonged periods of time in the postictal period, likely via brainstem and insula sites involved in chemosensation and interoception. They further provide insights into SUDEP, may help identify those at greatest risk, and may lead to treatments to prevent SUDEP.

## Graphical abstract

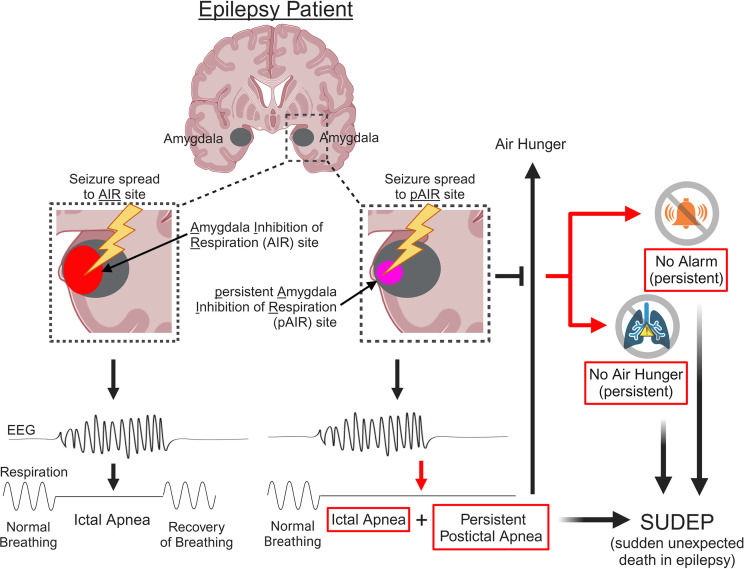

## Introduction

Sudden unexpected death in epilepsy (SUDEP) accounts for 10%–50% of all deaths in individuals with medically refractory epilepsy (1). Accumulating evidence indicates that the majority of SUDEP cases occur after seizures from loss of breathing (2, 3). Patients at greatest risk of SUDEP are those with pharmacoresistant epilepsy, especially those who are referred for epilepsy surgery and/or continue to have seizures after surgery (1, 4). Critical insights into SUDEP come from rare cases witnessed in epilepsy monitoring units where electroencephalographic, cardiac, and respiratory function could be assessed (3). In those cases, apnea (absence of breathing) was identified from video recordings of chest wall movement, which was visible after seizure activity ceased (i.e., in the postictal period) (3). In each case, transient episodes of postictal apnea persisted for minutes until the apnea became sustained and there was no recovery of respiratory movement prior to terminal asystole and death. Other studies of cardiorespiratory function following generalized convulsive seizures identified near-SUDEP cases (5–7) and patients who subsequently died of SUDEP (8). In these cases, periods of persistent postictal apnea and breaths with markedly smaller tidal volume occurred. In some cases, this led to temporary asystole that resolved spontaneously or required cardiopulmonary resuscitation. Together, these studies indicate that postictal apnea is a major cause of SUDEP. However, the mechanistic causes of postictal apnea are unknown.

In contrast to postictal apnea, apnea that only occurs during a seizure (i.e., ictal apnea) is more common but less severe than persistent postictal apnea and is estimated to occur in 33%–40.5% of all seizures (7, 9–11). In studies of adult and pediatric epilepsy patients undergoing intracranial electroencephalography (iEEG) and continuous respiratory monitoring, seizure propagation to the amygdala was associated with ictal apnea (12). Delivering electrical stimuli directly to a focal site in the amygdala with implanted electrodes reproduced the apnea observed during seizures (12–15), thus confirming a role for the amygdala in inhibiting respiration and causing apnea. However, these events were self-limited and breathing promptly resumed when the electrical stimulation or seizure activity within the amygdala ended. Together these studies suggest that the amygdala is functionally connected to the brainstem respiratory network, can inhibit breathing, and plays a key role in ictal apnea. However, they also raise questions about amygdala seizure–induced ictal apnea and its potential relationship with postictal apnea associated with SUDEP. If seizure activity in the amygdala stops, what causes postictal apnea? Thus, are these 2 forms of apnea (ictal vs. postictal) related? Is postictal apnea a more severe form of ictal apnea, or is it a distinct entity? Does postictal apnea occur only after generalized convulsive seizures (postconvulsive central apnea), or can postictal apnea also be a sequela of focal seizures? And what mechanisms, brain sites, and pathways might be involved in postictal apnea? Answering these questions is critical for improving our understanding of the neural mechanisms that lead to SUDEP. This knowledge is needed to inform the rational design of new SUDEP prevention management strategies.

To answer such questions and determine whether the amygdala or other forebrain sites might be involved in postictal apnea and possibly SUDEP, we studied 20 patients with intractable epilepsy undergoing iEEG monitoring for seizure focus localization. We recorded iEEG and breathing during stimulation-evoked seizures to identify brain sites associated with postictal apnea. We also functionally mapped the brain using electrical stimulation below seizure threshold to identify focal sites that elicited changes in breathing and characterize the nature and persistence of site-specific stimulation effects, including the persistent apnea observed in SUDEP. Machine learning was subsequently used to identify a common region across patients critical for postictal apnea. To understand mechanisms underlying these respiratory effects, we examined whether CO_2_ respiratory sensitivity was altered. We then used a novel experimental approach that combined direct electrical brain stimulation concurrent with functional magnetic resonance imaging (fMRI) to identify brain connections that underlie postictal apnea.

## Results

### Stimulation-induced seizures in the amygdala elicited ictal and persistent postictal apnea.

**Figure 1:**
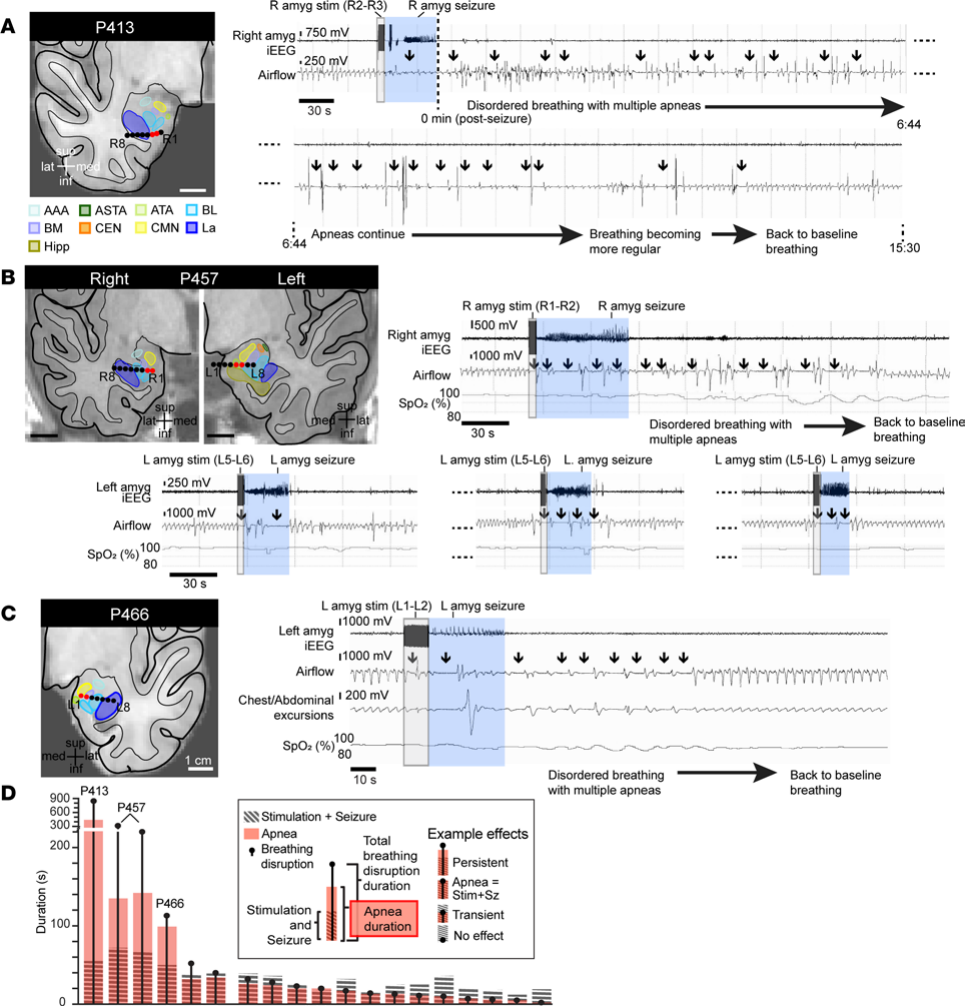
Stimulation-induced seizures in the amygdala evoked both ictal and persistent postictal apnea. (**A**) Anatomical localization of right amygdala depth electrode contacts (black circles) in the coronal plane of P413. Numbers 1–8 specify electrode contacts from medial to lateral. Amygdala nuclei are represented as follows: La, lateral nucleus (royal blue); BL, basolateral nucleus (light blue); BM, basomedial nucleus (lavender); CEN, central nucleus (orange); CMN, cortical and medial nuclei (yellow); ATA, amygdala transition areas (light green); ASTA, amygdalostriatal transition area (forest green); AAA, anterior amygdala area (aqua); Hipp, hippocampus (brown). Short stimulation (gray shading) of contacts R2–R3 (red circles) in the right amygdala of P413 induced a focal seizure (blue shading). This resulted in postictal apneas (arrows) that became more profound 2.5 minutes after seizure termination and persisted for over 13 minutes beyond seizure end. iEEG signal is shown on top, and respiratory traces below (inspiration plotted up; conventions remain the same for **B** and **C**). (**B**) Anatomical localization of right and left amygdala depth electrode contacts in the coronal plane of P457. Stimulating right amygdala contacts R1–R2 (red circles) induced apnea during stimulation and during an induced unilateral right amygdala seizure. Postictal apneas persisted for over 90 seconds. Stimulating contacts L5–L6 in the contralateral left amygdala induced apnea during stimulation and induced unilateral focal seizures (bottom 3 stimulation trials). Normal baseline breathing resumed almost immediately following seizure termination. (**C**) Anatomical localization of left amygdala depth electrode contacts in the coronal plane of P466. Stimulating contacts L1–L2 in the left amygdala induced apnea during stimulation and during an induced unilateral left amygdala seizure. Postictal apneas persisted for over 60 seconds. (**D**) Summary of all 19 seizures elicited by stimulation in 7 patients. Duration of stimulation plus seizure (hatched gray bars), total apnea time (red bars), and total disrupted breathing time (black dot and line) are shown for each seizure elicited by stimulation.

Looking for possible occurrences of postictal apnea (at least 20 seconds of apnea after seizure terminated), we systematically analyzed breathing before, during, and after stimulation-induced seizures in 20 patients while they were awake and resting in bed in the epilepsy monitoring unit. Importantly, none of these seizures required intervention, and thus respiratory recordings continued unimpeded while the patient was monitored alone in bed. Strikingly, in 3 patients, amygdala seizures (*n* = 4 seizures) not only elicited ictal apnea but led to persistent postictal apnea (Figure 1). In P413, 5-second stimulation of the right amygdala induced a 30-second focal seizure with ictal apnea, which was followed by disordered breathing and apneas in the immediate postictal period (Figure 1A). Notably, postictal apneas became longer and more profound approximately 2.5 minutes after the seizure terminated, a pattern that resembled previously reported cases of postictal apnea in SUDEP (3). Remarkably, postictal apneas lasted for more than 13 minutes beyond seizure termination before returning to a normal breathing pattern. Similarly, in a second patient (P457), stimulation of the right amygdala induced a focal seizure with apnea and intermittent sporadic breaths (Figure 1B). After the seizure activity ceased, the apneas persisted into the postictal period for 2.5 minutes, a finding reproducible in a second seizure induced from the right amygdala. Stimulating the contralateral amygdala induced unilateral seizures (*n* = 3 seizures) with only ictal apnea, as regular breathing resumed quickly following seizure termination (Figure 1B, bottom 3 panels). In a third patient (P466), amygdala stimulation evoked a seizure with ictal apnea and postictal apneas persisting for over 60 seconds (Figure 1C). After these events, all 3 patients denied any awareness that their breathing had changed and did not report any shortness of breath or air hunger or display any visible signs of respiratory distress during or after the postictal apneas. In 4 other patients, amygdala stimulation resulted in focal seizures (*n* = 12 seizures) that were associated with ictal apnea but not postictal apnea (Figure 1D and Table 1). Together, these findings indicate that seizure activity within the amygdala is associated with both ictal apnea and postictal apnea. Moreover, these findings suggest potentially important differences between patients in their vulnerability to the suppressive respiratory effects of seizure activity in the amygdala.

### Amygdala stimulation below seizure threshold evoked persistent post-stimulation apneas.

To test whether postictal apnea was due to inhibition of respiration as a result of seizure activity within the amygdala versus other forebrain sites, we functionally mapped sites for breathing effects using focal electrical stimulation below seizure threshold. In 2 of 20 patients (P413 and P457), stimulation-induced seizure thresholds were so low that electrical stimulation effects could not be distinguished from seizure-induced effects. In the 18 patients that we could map, apnea was observed in 16 patients during amygdala stimulation (Table 1). Stimulation at all other forebrain sites did not elicit apnea.

**Figure 2:**
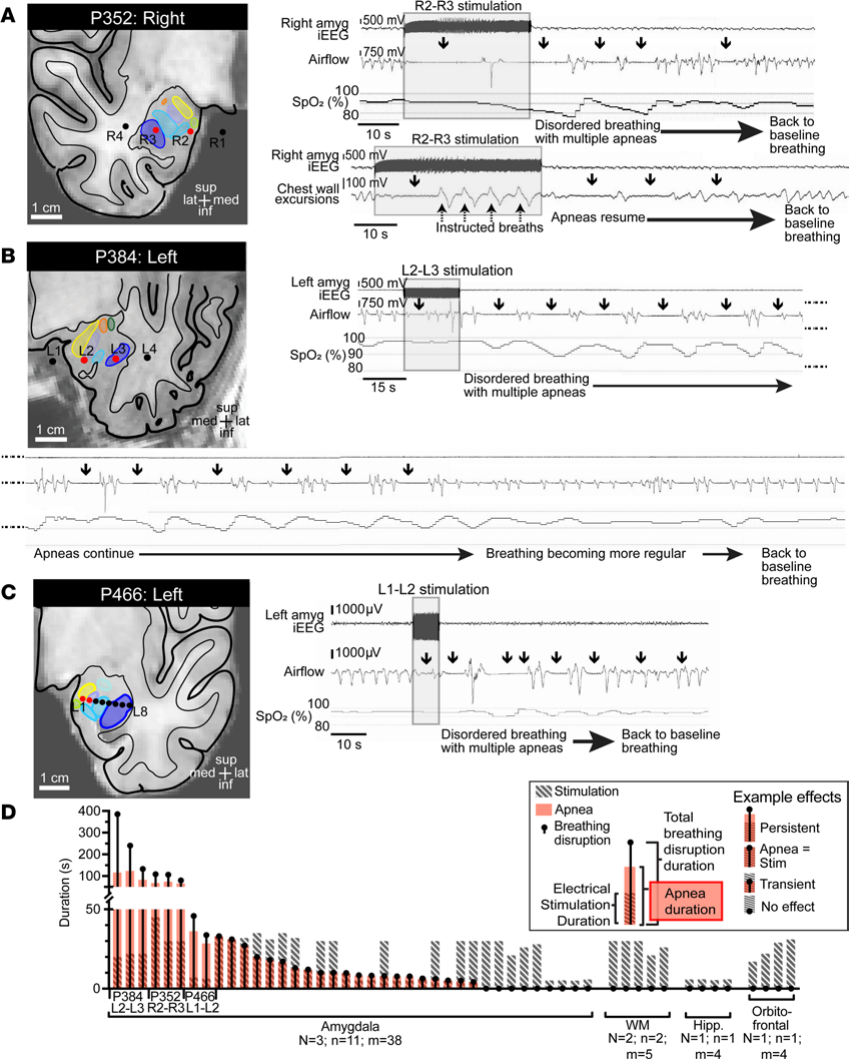
Electrical stimulation of the amygdala evoked persistent post-stimulation apnea, an effect not seen with stimulation outside the amygdala. (**A**) Anatomical localization of right amygdala depth electrode contacts (black circles) in the coronal plane of P352. Amygdala nuclei are defined as in Figure 1 (schematic remains the same for **B** and **C**). Stimulating contacts R2–R3 (red circles) in P352 resulted in apnea that was almost immediate in onset and lasted the duration of stimulation. After stimulation ended, apneas (black arrows) persisted in total for nearly 60 seconds. Repeated intervals of decreased oxygenation were observed with apneic periods. P352 was able to override stimulation-induced apnea through instructed voluntary breathing, but still exhibited post-stimulation apneas. iEEG signal is shown on top and respiratory trace from nasal pressure transducer shown below (inspiration plotted up; duration of stimulation depicted by shaded gray box; conventions remain the same for **B** and **C**). (**B**) Stimulating contacts L2–L3 in the left amygdala of P384 also resulted in post-stimulation apneas that lasted over 100 seconds in total and a total breathing disruption time of 5 minutes after stimulation ended. (**C**) Post-stimulation apneas were also observed with stimulation of contacts L1–L2 of the left amygdala in P466. (**D**) Summary of all stimulation trials (*n* = 51) for P352, P384, and P466, showing duration of stimulation (hatched gray bars), total apnea time (red bars), and total disrupted breathing time (black dot and line). Stimulation of amygdala sites in **A**–**C** led to persistent post-stimulation apneas with every trial at those sites, whereas amygdala stimulation outside these sites led to apnea of various degrees, lasting the duration of stimulation to no effect. Stimulation with the same amplitude (10–15 V) and frequency (50 Hz) outside the amygdala (white matter, WM; hippocampus, Hipp; and orbitofrontal sites) failed to induce any apnea.

Strikingly, in 3 of the 16 patients, P352, P384, and P466 (Figure 2 and Table 1), electrical stimulation of the amygdala below seizure threshold not only induced apnea during stimulation but also elicited apnea after stimulation (at least 20 seconds of apnea after electrical stimulation had been terminated; Figure 2). Post-stimulation apnea was consistently observed across multiple trials in each patient. Importantly, post-stimulation apneas occurred in the absence of seizure activity and had characteristics that were remarkably similar to the postictal apnea spells observed following seizures described above. For example, in P352, stimulating the right amygdala below seizure threshold for 30 seconds led to post-stimulation apneas (Figure 2A) lasting 62.9 seconds on average over 3 trials. P352 was able to override the stimulation-induced apnea when instructed to breathe, confirming that apnea was not caused by impairment in respiratory musculature or airway obstruction (e.g., due to laryngospasm) (Figure 2A). In the absence of instruction to take additional breaths, apnea resumed, suggesting that cortically controlled breathing did not entrain or re-engage the brainstem central pattern generator responsible for automatic breathing (i.e., breathing not driven by conscious volition or by a ventilator). In P384, a 12.5-second left amygdala stimulation induced a period of apnea that was followed by repeated apneic periods for over 100 seconds after stimulation ended (Figure 2B). In P466, persistent apneic periods lasted up to 45.7 seconds after the end of a 7-second left amygdala stimulation (Figure 2C), an effect similar to the postictal apnea observed in the same patient (Figure 1C). These findings indicate that in some patients with medically refractory epilepsy, electrical stimulation of the amygdala can persistently inhibit breathing and cause apneas that extend beyond the window of stimulation.

### The amygdala was the only stimulated forebrain site that elicited post-stimulation apnea.

In total, 51 stimulation trials were conducted at 15 sites in the 3 patients with post-stimulation apnea (Figure 2D). As described above in each of the 3 patients, stimulation of specific amygdala sites elicited post-stimulation apneas. However, stimulation of 8 other amygdala sites across 30 trials in these patients did not induce post-stimulation apneas but elicited a range of effects on breathing, from no change to apnea that lasted the duration of stimulation. In contrast, stimulating 4 non-amygdala sites in adjacent white matter, hippocampus, and orbitofrontal cortex across 14 trials had no effect on breathing, either during or after stimulation. These findings suggest that post-stimulation apnea was limited to amygdala stimulation in this cohort of patients and brain sites studied here and not from widespread current spread to adjacent sites. Moreover, only specific sites within the amygdala were sufficient to induce post-stimulation apnea.

### Amygdala stimulation evoked persistent post-stimulation apneas without air hunger or distress.

Because our patients denied awareness of breathing changes and did not report any air hunger during postictal apnea, we asked whether these sensations were also lacking during post-stimulation apnea. During post-stimulation apnea, all 3 subjects were unaware that their breathing had changed, did not struggle to breathe at any time, did not report an urge to breathe, maintained a neutral facial expression, did not report any emotional change, and denied experiencing any sense of respiratory distress. The lack of these sensations associated with post-stimulation apnea indicates that the amygdala not only caused respiratory arrest, but also suppressed dyspnea and the primal sensation of air hunger associated with apnea. Furthermore, it suggests that in some patients with epilepsy, the normal protective perception of air hunger may be suppressed for an extended period of time following a seizure.

### Persistent postictal and post-stimulation apnea localized to a specific site in the amygdala.

**Figure 3:**
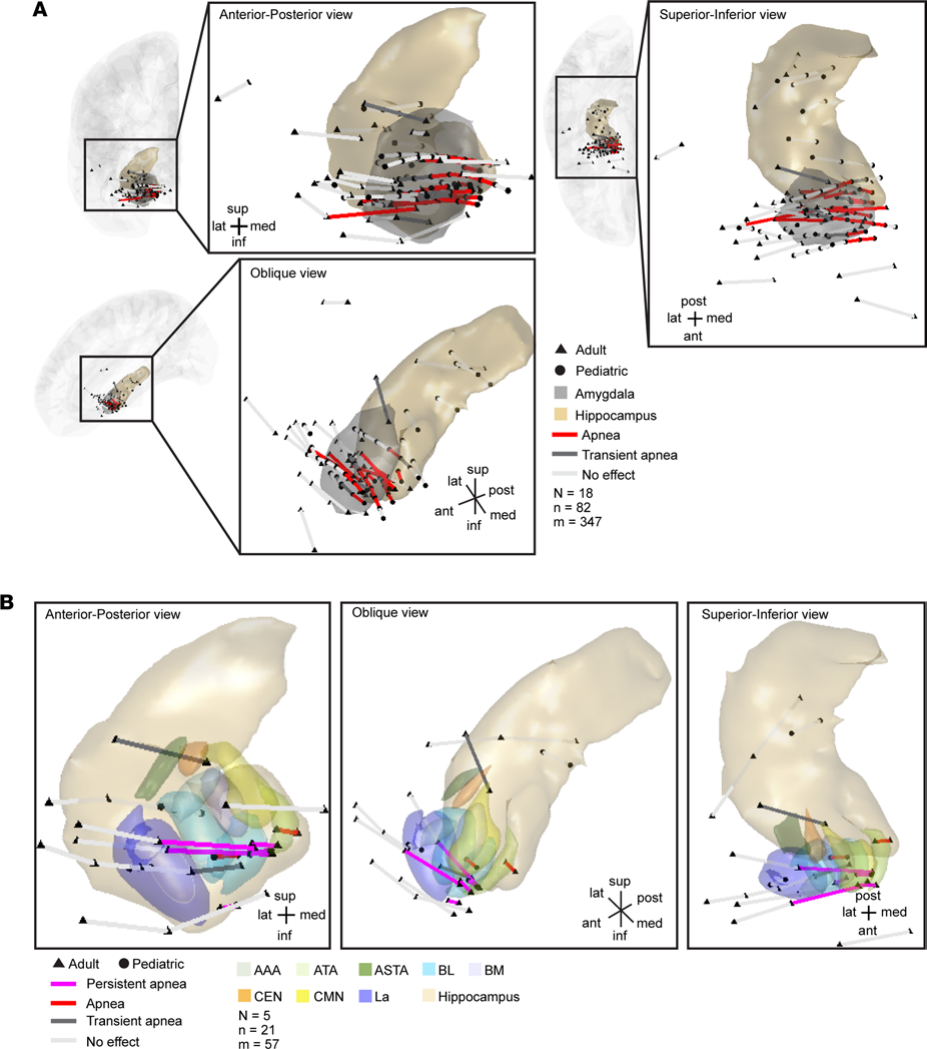
Across-subject analysis localized post-stimulation apnea and postictal apnea to a specific site in the amygdala. (**A**) Anterior-posterior, superior-inferior, and oblique views of all stimulated electrode pairs (*n* = 82 sites) in the temporal lobe and inferior frontal lobe across 18 subjects who had stimulation below seizure threshold (adult: triangles; pediatric: circles) plotted in a common coordinate system (MNI). Electrode contact pairs that produced apnea (red lines) were located in the medial amygdala. Electrode contact pairs that produced transient apnea (dark gray lines) were typically located just lateral or adjacent to this medial region. Electrode contact pairs that failed to induce apnea (light gray lines) were located in the lateral amygdala, areas just outside the amygdala, in hippocampus, Heschl’s gyrus, and orbitofrontal cortex. Electrode contacts may appear outside of the template brain due to anatomical variation across subjects relative to the MNI coordinate system. All electrode contacts were plotted in the right hemisphere for simplicity because no differences were observed between right and left amygdala stimulation. (**B**) Anterior-posterior, oblique, and superior-inferior views of all stimulated electrode pairs in the amygdala and hippocampus across the 5 subjects with persistent apnea, plotted in a common coordinate system (MNI). Electrode pairs that induced persistent post-stimulation and postictal apneas are denoted by magenta lines and clustered together mostly spanning the basolateral nucleus and including the cortical and medial nuclei and the medial aspect of the lateral nucleus. Electrode pairs that induced apnea are denoted by red lines, and transient apnea sites are denoted by dark gray lines; sites that did not induce apnea are depicted in light gray. See Supplemental Table 1 for a list of MNI coordinates and the respiratory effect for each contact pair. Nuclei are color-coded with the same conventions as in Figure 1.

Because persistent post-stimulation apnea did not occur with stimulation at every site in the amygdala and because the amygdala is composed of multiple nuclei with different efferent and afferent connections, we asked whether persistent apnea localized to an amygdala site and specific amygdala nuclei. We plotted all stimulated electrode pairs within the amygdala and immediately adjacent to the amygdala, including the hippocampus, orbitofrontal cortex, Heschl’s gyrus, and adjacent white matter, for all subjects onto a 3D common anatomical space using the Montreal Neurological Institute (MNI) coordinate system. We projected the respiratory effects elicited from each electrode pair onto the 3D anatomical common space. Because of the dense number of contacts stimulated, we first plotted the results from the 18 patients we could functionally map excluding sites causing persistent apnea so that our findings could be more clearly illustrated (*N* = 18 patients, *n* = 82 sites, *m* = 347 stimulation trials; Figure 3A). Apnea occurred throughout the duration of stimulation at 25 amygdala sites. Transient apnea, in which breathing resumed prior to the end of stimulation, occurred at 10 amygdala sites. Stimulation at 47 sites within the amygdala or in structures near the amygdala elicited no effect on breathing. Stimulations that induced apnea localized to a medial region of the amygdala. This location was similar across all ages (pediatric and adult), appearing consistent with the previously identified amygdala inhibition of respiration (AIR) site (13).

Next, we projected the results from the 5 patients who had persistent post-stimulation and postictal apnea (*N* = 5 patients, *n* = 21 sites, *m* = 57 stimulation trials; Figure 3B) onto the 3D anatomical common space. Persistent apnea was elicited at 5 sites, apnea limited to the duration of stimulation occurred at 2 sites, transient apnea occurred at 2 sites, and 12 sites had no effect. Sites that induced persistent apnea clustered together and mostly spanned the basolateral amygdala but included the medial portion of the lateral nucleus and corticomedial nuclei. This effect was specific to these nuclei and was not seen with stimulation of other immediately adjacent sites in the lateral amygdala, white matter immediately lateral to the amygdala, or hippocampus.

### Machine learning predicted an amygdala site that could induce persistent postictal or post-stimulation apnea.

**Figure 4:**
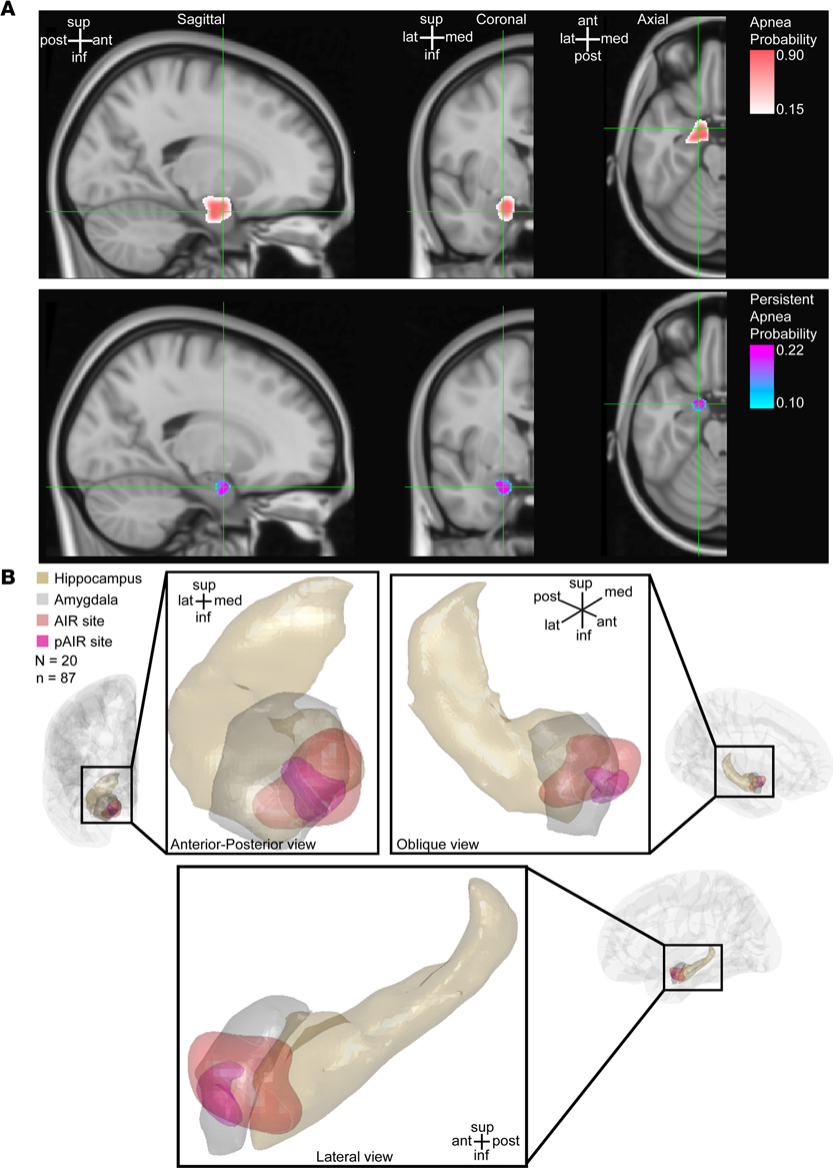
Machine learning algorithm identified a site in the amygdala critical for persistent postictal apnea. (**A**) Probability map of apnea (top) and persistent apnea (bottom) resulting from support vector machine classification of respiratory effects predicted from MNI coordinates of 87 stimulated electrode contact pairs across 20 subjects (Supplemental Table 1). (**B**) The persistent amygdala inhibition of respiration (pAIR) site (magenta) predicts persistent post-stimulation and postictal apneas based on the results of **A**, overlaid on amygdala (gray, FSL) and hippocampus (brown, FreeSurfer) (68, 69) in anterior-posterior, oblique, and lateral views. The pAIR site is located in a subregion of the AIR site (red). Probability map is plotted in the right hemisphere only for simplicity because no systematic differences were observed between right and left amygdala stimulation, and all left-sided contacts were projected to the contralateral hemisphere for the purpose of classification. For simplification, the results for transient apnea are not shown.

Next, we asked whether the region in the amygdala that induced persistent apnea was different from the region in the amygdala that induced apnea. We used machine learning classification to predict stimulation effects from sites. We applied a multi-class support vector machine classifier to predict the respiratory outcome (persistent apnea, apnea, transient apnea, or no effect) based on the MNI coordinate of the midpoint between stimulated electrode contacts (13) across this 20-patient cohort. This included results from stimulation with or without associated seizures (*N* = 20 patients, *n* = 87 sites) to create probabilistic maps of the respiratory outcome. Probabilistic maps of the respiratory outcome identified a small region of the amygdala that was most likely to result in apnea (up to a 90% probability) (Figure 4, A and B), a focal site consistent with the previously identified AIR site. Persistent apnea, although not observed in all subjects or all amygdalae, only occurred with stimulation in a more focal area of the amygdala. This focal site with the highest likelihood of persistent apnea lies within a circumscribed area of the AIR site (Figure 4, A and B), which we refer to as the persistent AIR (pAIR) site.

### Amygdala stimulation evoked persistent post-stimulation hypoventilation despite hypercapnia.

**Figure 5:**
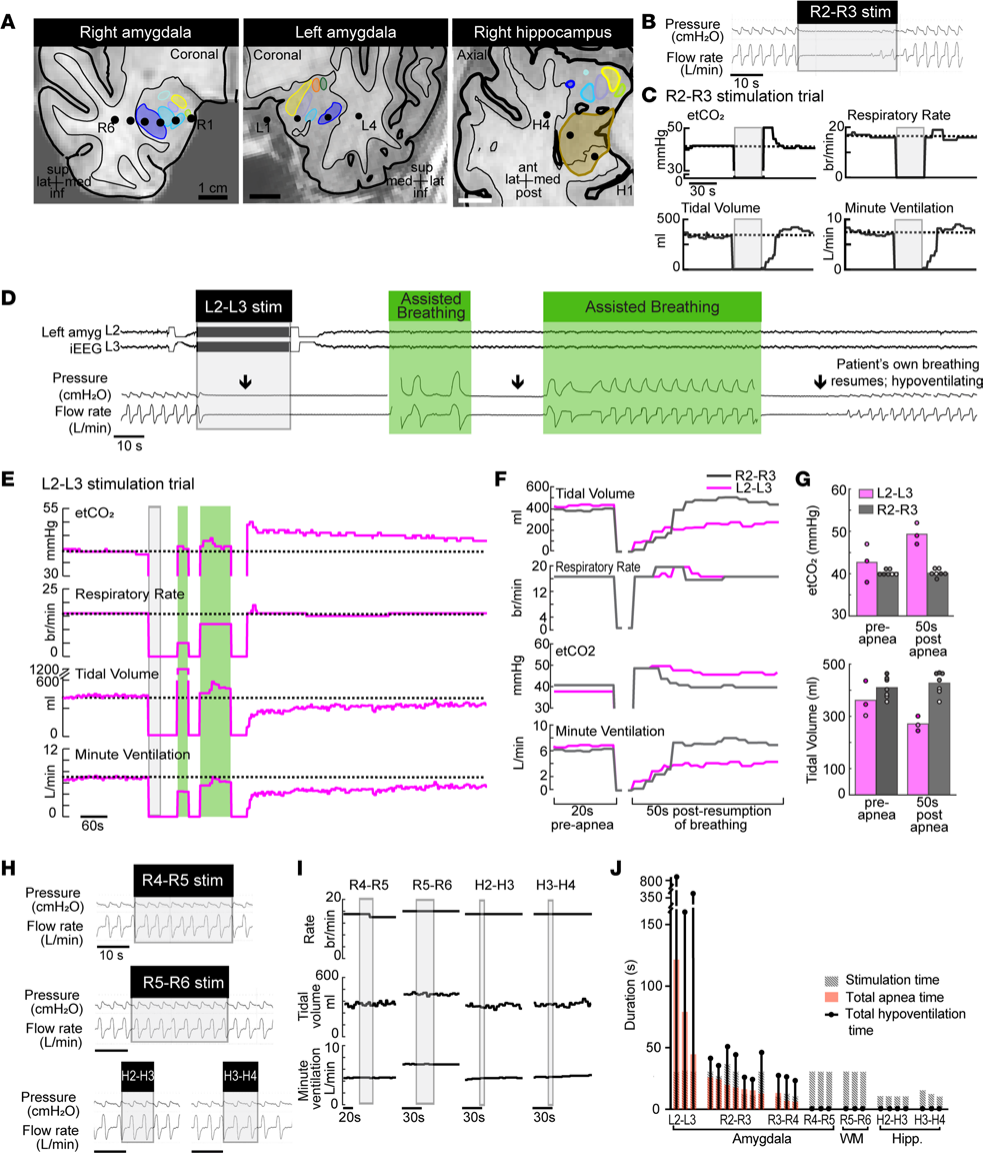
Amygdala stimulation evoked persistent post-stimulation hypoventilation despite hypercapnia. (**A**) Electrode contacts superimposed upon P384’s temporal lobes (see Figure 1A). (**B**) While the patient was under anesthesia, intubated, and breathing independently, R2–R3 stimulation induced apnea during stimulation (as was observed at bedside). (**C**) etCO_2_ increased after apnea followed by rapid increase in respiratory rate (RR), tidal volume (TV), and minute ventilation (VE) to normalize CO_2_ levels. Dotted line indicates average pre-stimulation values. (**D**) L2–L3 stimulation induced long-lasting inhibition of independent breathing, causing post-stimulation apneas. Manual breaths and ventilator-dependent breathing were provided without difficulty (green shading) but did not initiate independent breathing. (**E**) Once independent breathing resumed, baseline RR resumed, but etCO_2_ remained elevated and TV and VE decreased below baseline. Thus, P384 had persistent hypoventilation despite elevated etCO_2_ for more than 10 minutes after stimulation. During this time, both etCO_2_ and TV slowly returned toward baseline. (**F**) Comparison of respiratory measurements before and after apnea from R2–R3 (dark gray) and L2–L3 (magenta) stimulation. Site L2–L3 resulted in prolonged hypoventilation with elevated etCO_2_ and lower TV and VE after independent breathing resumed compared with R2–R3. (**G**) Average ventilatory values before and 50 seconds after breathing resumed from stimulation of R2–R3 (*m* = 7) and L2–L3 (*m* = 3). etCO_2_ was higher but TV was lower for L2–L3 50 seconds after independent breathing resumed, indicating persistent hypoventilation after L2–L3 stimulation. (**H** and **I**) Lateral amygdala (R4–R5), adjacent white matter (R5–R6), and hippocampus (H2–H3, H3–H4) stimulation failed to induce apnea or abnormal breathing (**H**) or changes in RR, TV, or VE (**I**). (**J**) Summary of all trials (*m* = 26) under anesthesia for P384. Only stimulation of L2–L3 led to persistent post-stimulation apneas. Stimulation in nearby white matter and hippocampus with the same parameters (10–15 V; 50 Hz) failed to induce apnea.

Because previous studies suggested that postictal hypoventilation in the absence of complete respiratory arrest may also play a role in SUDEP (8, 16), we explored whether amygdala stimulation might alter and suppress the normal respiratory response to elevated levels of CO_2_. We used a unique opportunity to study P384 in the operating room while the patient was under anesthesia, orally intubated, breathing on his own, and connected to a ventilator. This clinical setting enabled us to obtain precise measures of tidal volume, minute ventilation, and end-tidal CO_2_ (etCO_2_) (Figure 5). Stimulation at amygdala sites across multiple trials induced apnea similar to what was observed when P384 was awake. For example, a 30-second stimulation of the right amygdala (R2–R3, Figure 5A, left panel) induced apnea (Figure 5B). This apnea resulted in an increase in etCO_2_ above baseline levels (hypercapnia) (Figure 5C). After stimulation ended, tidal volume, respiratory rate, and minute ventilation rapidly increased above baseline levels, consistent with increased CO_2_ levels as a potent driver of respiration. Importantly, these findings indicated that the drive to breathe was intact while the patient was under anesthesia. Increased ventilation was followed by a reduction in etCO_2_ and a subsequent return to normal ventilation levels within 30 seconds. In stark contrast, a 30-second stimulation of the same left amygdala contacts (L2–L3, Figure 5A, middle panel) that caused post-stimulation apnea while P384 was awake induced apneas that persisted more than 30 seconds after stimulation ended and lasted 153 seconds total (Figure 5D). However, unlike with the previous stimulations, after independent breathing resumed, tidal volumes and minute ventilation remained low for more than 15 minutes, despite the patient being hypercapnic (Figure 5, E–G). As the tidal volume slowly increased toward baseline levels, the hypoventilation resolved, and etCO_2_ slowly returned to baseline levels. Stimulations of a more lateral amygdala site (Figure 5A, left panel), adjacent white matter (Figure 5A, left panel), and hippocampus (Figure 5A, right panel) did not cause apnea or hypoventilation (Figure 5, H and I), suggesting that these effects were amygdala specific and localized to a specific subregion of the amygdala (Figure 5J). These findings suggest that stimulating a specific amygdala site reduced the CO_2_ respiratory drive, and that reduced CO_2_ sensitivity was not due to an inherent genetic trait (8) or anesthesia (17). Moreover, these findings suggest that ictal and postictal hypoventilation may be due to seizure activity in the amygdala that reduces CO_2_ respiratory drive.

### Persistent postictal apnea and post-stimulation apnea were not due to ongoing seizure activity or other neural correlates in the amygdala.

**Figure 6:**
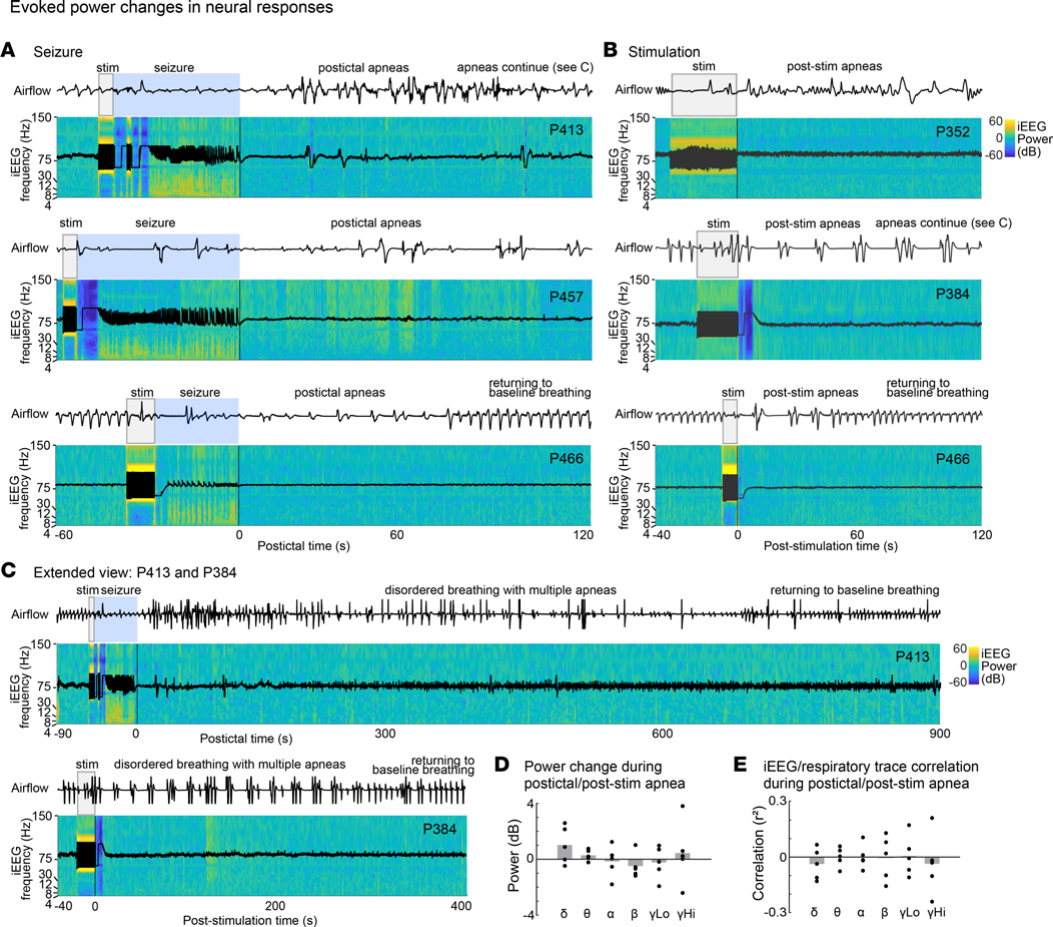
Persistent postictal apnea and post-stimulation apnea were not due to ongoing seizure activity or other correlated neural activity in amygdala. (**A**) Time-frequency representation of the peri-ictal period for subjects P413, P457, and P466. iEEG during seizure trials indicates an increase in power (warmer colors) during the seizures but no consistent changes in the spectrotemporal response properties in the persistent apnea period. Respiratory trace shown above time-frequency plots identifies the apneas and disrupted breathing from stimulation-evoked seizure. (**B**) Stimulation without seizure also shows no consistent spectrotemporal changes associated with the post-stimulation apneas. (**C**) Extended view of 2 subjects who had persistent apneas longer than 5 minutes, P413 and P384. No changes were observed over the entire period of persistent postictal and post-stimulation apneas. (**D**) iEEG power changes before versus during postictal or post-stimulation apneas. Each dot represents a value from 1 patient; gray bars indicate means across patients. No significant power changes were seen in any of the canonical iEEG frequency bands (delta 1–4 Hz, theta 4–8 Hz, alpha 8–12 Hz, beta 13–30 Hz, low gamma 30–80 Hz, high gamma 80–150 Hz). (**E**) No significant correlation between the respiratory signal and the envelope of each canonical EEG band was observed. Each dot represents a value from 1 patient; gray bars indicate means across patients.

Next, we asked whether postictal or post-stimulation apnea was due to persistent seizure or other correlated electrophysiologic activity in the amygdala (Figure 6). In all 5 patients, iEEG analysis did not reveal any amygdala seizure events associated with postictal or post-stimulation apneas (Figure 6, A–C). Analysis of canonical iEEG frequency bands (delta, theta, alpha, beta, low gamma, high gamma) from amygdala recordings obtained during the periods of postictal and post-stimulation apnea compared with normal baseline breathing did not identify any significant power changes (Wilcoxon’s signed-rank test; *P* > 0.625, FDR corrected; Figure 6D). Next, we examined the correlation between the respiratory trace and the envelope of the canonical EEG frequency bands to determine whether fluctuations in amygdala activity were related to the patient’s ventilatory patterns. No difference in correlation was observed between states of normal baseline breathing and states of postictal or post-stimulation apneas (Wilcoxon’s signed-rank test; *P* > 0.7, FDR corrected; Figure 6E). These findings suggest that postictal and post-stimulation apnea was not due to persistent seizure activity or correlated electrophysiologic activity in the amygdala but due to persistent suppressive effects at downstream brainstem sites engaged in regulating breathing.

### The pAIR site is functionally connected to pontomedullary brainstem and insula sites.

**Figure 7:**
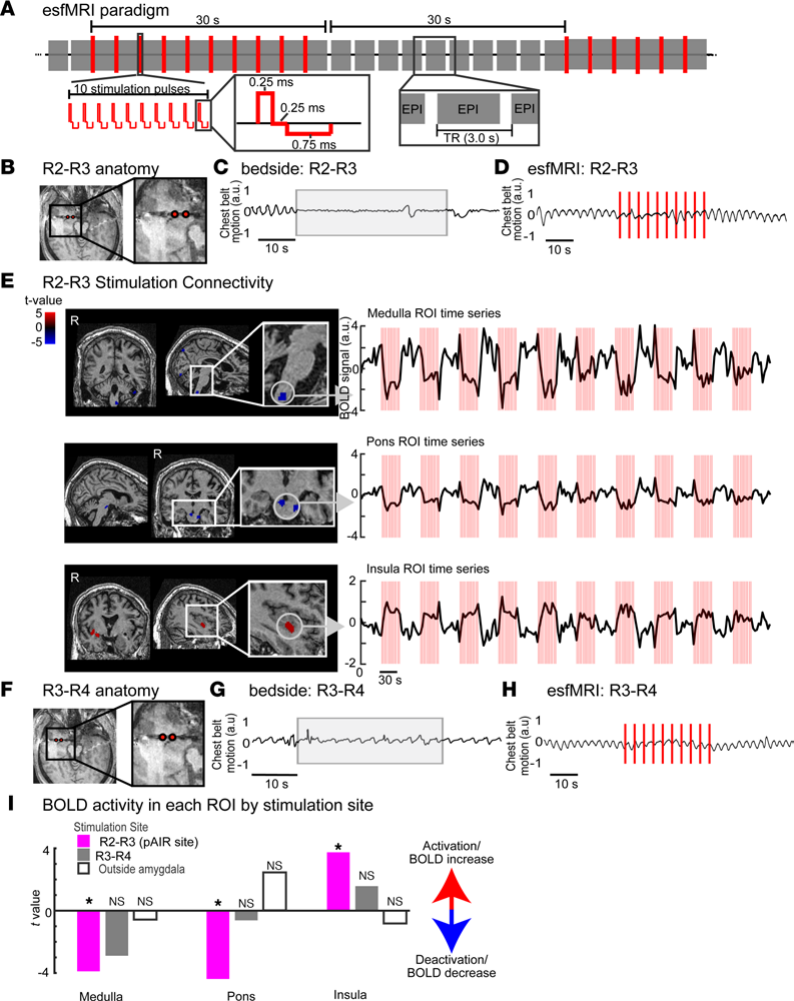
Amygdala is functionally connected to the pons, medulla, and insula. (**A**) Schematic of electrical stimulation concurrent with functional MRI paradigm (es-fMRI; adapted from Rocchi, Oya, and colleagues, ref. 70). EPI, echo planar imaging; TR, repetition time. (**B**) Axial MRI of P352’s bilateral temporal lobes with zoomed view of right amygdala. Stimulated contacts R2–R3 (red circles) are located within the pAIR site. (**C**) Continuous stimulation of R2–R3 at bedside (light gray shading) induced persistant apnea. (**D**) During es-fMRI, the same site was stimulated with stimulation pulses (red lines) with some disruption to the subject’s normal breathing. (**E**) BOLD response associated with stimulation of site R2–R3 in P352. Stimulation of the R2–R3 site caused a significant decrease of BOLD activity within the medulla (*t* value = 3.89, *P* < 0.001; top panel) and superior part of the pons (*t* value = 3.85, *P* < 0.001; middle panel). Stimulation of the pAIR site significantly increased BOLD activation in the ventral part of the insula (*t* value = 3.74, *P* < 0.001; bottom panel). (**F**) Axial MRI of P352 with zoomed view of the right amygdala and anterior temporal cortex. Stimulated contacts R3–R4 are shown with red circles. (**G**) Stimulation of this site at bedside (light gray) was not associated with changes in breathing. (**H**) Stimulation during es-fMRI caused minimal or no changes in breathing. (**I**) Comparison of BOLD activity in each ROI by stimulation site. R2–R3 stimulation (pAIR site, magenta) significantly decreased BOLD activity in the medulla and pons while increasing BOLD activity in the ventral insula. In contrast, stimulation in the amygdala but outside the pAIR site and AIR site (dark gray) revealed no significant BOLD changes in the medulla or pons. Stimulation outside the amygdala in the contralateral left insula was used as a control site (white) and did not result in significant BOLD changes in the brainstem. *P < 0.05.

To identify sites downstream from amygdala that may be critical for ictal and postictal apnea without air hunger, we used a novel experimental approach, electrical stimulation fMRI (es-fMRI) (18). With this approach, electrical stimuli are delivered through depth electrode contacts while the patient is in the MRI machine. The electrical stimulation can activate or inhibit functionally connected sites elsewhere in the brain, identified by measurement of the blood oxygen level–dependent (BOLD) responses within functionally connected sites (Figure 7A). Using the es-fMRI method, it is feasible to study effective connectivity, defined as causal connections between brain sites, as opposed to simple correlative associations (19). First used in nonhuman primates, major recent technical advances have been made and obstacles overcome for use of this experimental method in humans. With es-fMRI, we can study causal effects of amygdala stimulation on the whole brain including brainstem, with a degree of brain coverage not possible with iEEG. 

Because of limitations associated with performing MRI-based studies in younger patients (e.g., requirement for sedation), and practical considerations related to patient care workflow, not all implanted patients involved in brain physiology experimental studies participate in this novel experimental approach. Despite these limitations and constraints, one of our patients with persistent apnea, P352, was able to participate in the es-fMRI protocol with stimulation of the pAIR site (amygdala contacts R2–R3, Figure 7B). Unlike continuous electrical stimulation at the bedside, which caused persistent apnea (Figure 7C), an effect that would confound fMRI BOLD analysis, short stimulation pulses of the pAIR site in P352 only briefly disrupted normal breathing (Figure 7D). The periods of respiratory disruption that occurred during the es-fMRI experiment were too brief to substantially alter CO_2_ concentrations, which otherwise might have confounded the fMRI BOLD analysis. Strikingly, stimulation caused a significant decrease in BOLD activity within the medulla (*P* < 0.001; Figure 7E, top panel) and superior pons (*P* < 0.001; Figure 7E, middle panel), brain sites known to be engaged in controlling respiration. Interestingly, we also found that stimulating the pAIR site significantly increased BOLD activation within the ventral insula (*P* < 0.001; Figure 7E, bottom panel), a site implicated in the perception of air hunger (20). 

To test whether the decreased BOLD activity in the medulla and pons was specific to the pAIR site stimulation, we also stimulated amygdala contacts R3–R4, which lie outside of this region (Figure 7F). R3–R4 stimulation produced no effects on breathing (Figure 7, G and H) and no significant changes in brainstem BOLD activity (Figure 7I, gray bars), suggesting that the es-fMRI BOLD responses in pons and medulla were specific to the R2–R3 sites. We further stimulated a control site outside the amygdala and found no effect on breathing or brainstem BOLD signal (Figure 7I, white bars). These findings suggest that apnea caused by amygdala stimulation at the pAIR site may be due to inhibition of the brainstem respiratory network in the medulla or pons, while suppression of air hunger may be related to stimulation-induced changes observed within the insula.

## Discussion

Here we used a multimodal approach to study 20 epilepsy patients at highest risk for SUDEP undergoing iEEG for seizure focus localization. Our experiments were designed to address several questions related to forebrain control of breathing, postictal apnea, and SUDEP. First, we asked whether ictal apnea and postictal apnea are related or are distinct, separate phenomena. Our data here suggest that they are related. Postictal apnea was preceded by ictal apnea in all cases, suggesting that ictal apnea may lead to postictal apnea, or that postictal apnea may be a severe form of ictal apnea that persists after seizure. Importantly, because we observed postictal apnea to follow ictal apnea in every case, these findings suggest that ictal and postictal apnea share underlying mechanisms.

Next, we asked whether postictal apnea can occur with focal seizures or whether it is a phenomenon that only occurs with generalized convulsive seizures. Our data here suggest that generalized convulsive seizures are not required for postictal apnea. Others have hypothesized that ictal apnea results from seizure-induced cortical dysfunction, whereas postictal apnea only results from a generalized convulsive seizure that spreads to the brainstem and disrupts the brainstem respiratory network (7). Here we found that focal seizures were sufficient to induce both ictal and postictal apnea, indicating that focal seizures can disrupt the brainstem respiratory network even after the seizure ends. Although our data indicate that focal seizures can cause postictal apnea, accumulating evidence indicates that the majority of SUDEP cases occur from postictal apnea after a generalized convulsive seizure (3). Postictal apnea may be more likely to lead to death when an epilepsy patient is obtunded, confused, and their breathing becomes mechanically obstructed, a setting that is more likely to occur after a generalized convulsive seizure than a focal seizure.

Third, we asked what brain sites, pathways, and mechanisms might be involved in postictal apnea. Using invasive intracranial seizure recordings, we found that seizure activity in the amygdala evoked postictal apnea that persisted, lasting up to 13 minutes in one patient. These findings are consistent with the postictal apneas that occurred in monitored SUDEP cases in MORTEMUS (3). In MORTEMUS, postictal apneas varied, lasting up to 10 minutes after a generalized convulsive seizure while the patients were prone face down in bed and obtunded, leading to terminal apnea and death. Despite the importance of postictal apnea, no previous reports studied or reported postictal apnea during forebrain mapping studies of breathing control (12, 13, 15, 21–23). It is possible that other sites outside the amygdala may also be involved in postictal apnea, which would require study beyond the current work. Nevertheless, and notably, the present study is, to our knowledge, the first to identify a brain site that plays a critical role in postictal apnea in pediatric and adult epilepsy patients.

Electrical stimulation mapping combined with machine learning indicated that apnea persisting beyond the window of stimulation was evoked within a focal subregion of the amygdala (pAIR site) in our patient cohort. This pAIR site lies within the previously identified AIR site wherein apnea occurred during stimulation. This amygdala subregion specificity is consistent with the results of previous studies in which both seizures and electrical stimulation that did not involve the amygdala failed to cause ictal apnea or stimulation-induced apnea (12–14, 21). Through its role in inhibiting breathing and causing apnea, the amygdala might serve an important function in defense against threats, for airway protection, during speech, and/or for general volitional control of breathing (24, 25).

Using es-fMRI, the innovative approach that maps fMRI BOLD responses to electrical stimulation, pAIR site stimulation altered BOLD activity in the pons and medulla and in the ventral insula. These findings support important functional connections between the amygdala and sites previously implicated in respiratory control and interoceptive processing. Although still debated (26), converging evidence from a number of studies (27–30) suggests that reduction of BOLD activity is a marker for reduced neural activity. Thus, the decreased BOLD activity in the pons/medulla may suggest decreased activity in pontomedullary neurons, some of which may be critical for automatic respiratory rhythm generation such as those in parabrachial nuclei or the pre-Bötzinger complex (preBötC) (25, 27–32). Previous studies in rodents identified monosynaptic projections from the amygdala to preBötC neurons (33). Notably, inhibitory input to preBötC neurons has been reported to produce an extended refractory period (34). Thus, preBötC neuron inhibition may be particularly vulnerable to an extended period of suppression that might delay resumption of breathing. Alternatively, the reduced respiratory sensitivity to CO_2_ that followed amygdala stimulation may implicate CO_2_-sensitive neurons in the retrotrapezoid nucleus and serotonergic neurons in the medulla (35–38). Extended suppression of these neurons might interfere with the potent ability of CO_2_ to promote breathing. Thus, it has been suspected that impaired CO_2_ sensitivity might contribute to SUDEP (8, 16). These CO_2_-sensitive neurons stimulate preBötC neurons (37, 39, 40), an action thought to be especially important when CO_2_ levels rise and in non-wakeful periods such as sleep and unconscious postictal states (8, 41). Thus, seizure activity in the amygdala might lead to postictal apnea and SUDEP by inhibiting diverse cell types involved in breathing control.

When breathing is impaired, rising CO_2_ also serves as a powerful interoceptive stimulus triggering the primal sensation of air hunger, which gives rise to fear, arousal, and alarm (42). We have consistently found that patients with apnea during and after amygdala stimulation lack air hunger, the emotional response to air hunger, and even the conscious awareness that they have stopped breathing, despite increased CO_2_ levels (12, 13). The insula, which is known to be engaged in processing a range of interoceptive signals, is thought to play an important role in CO_2_-evoked air hunger (20, 43). Thus, it is noteworthy that pAIR site stimulation increased BOLD activity in the ventral insula, suggesting a possible mechanism for how amygdala stimulation suppresses air hunger. Although it is not immediately clear how increased activity in the insula would suppress the perception of air hunger, insula activation detected by BOLD activity might correlate with a disruption of normal patterns of local neural circuitry signaling. Conceivably, the increased BOLD activity might be dominated by activation of inhibitory interneurons (44). Supporting this finding, connections between the amygdala and insula have been well established, providing a clear anatomical rationale for these results (45). Thus, we speculate that loss of air hunger accompanying postictal apnea might be explained, at least in part, by prolonged disruption of insular function.

In this report, we found that only 5 of 20 patients studied developed postictal or post-stimulation apnea. Thus, our data suggest that some patients with medically intractable epilepsy may be more prone to developing postictal apnea. The findings also suggest that within a given patient, seizure activity in one amygdala may be more likely to induce postictal apnea than the contralateral amygdala, although there was not a consistent right or left laterality detected. It also remains possible that these differences between patients and within patients may be artificial. For clinical purposes, only a single depth electrode is placed in the amygdala in each patient, and thus not all subregions of the amygdala can be selectively mapped. Thus, if we had been able to thoroughly map every amygdala site, we might have evoked post-stimulation or postictal apnea in every patient. However, an alternative and, we believe, highly plausible explanation is that there are functional differences in the amygdala across subjects, due to developmental differences or acquired changes due to seizures. Changes in brain connectivity in patients with temporal lobe epilepsy have been reported, including between the amygdala and other brain areas (46–49). Moreover, developmental functional differences between the right and left amygdala have also been observed (50). Conceivably, these developmental and acquired mechanisms may explain observed differences between patients and between the right and left amygdala in individual patients.

This study has limitations that deserve consideration. First, the sample size is restricted because only a small subset of epilepsy patients are indicated for or elect to undergo intracranial electrode implantation (51). Moreover, only a subset of those implanted are indicated for or elect to participate in respiratory-related research protocols including es-fMRI. Second, SUDEP typically occurs when epilepsy patients experience a spontaneous seizure while alone, but all the patients examined in this study were under close observation within an epilepsy monitoring unit during seizures evoked by stimulation. Third, we cannot target specific neurons and activate or inhibit certain circuits with electrical stimulation. Thus, it is possible that apnea was evoked by spread of electrical current to immediately adjacent sites or white matter tracts. However, with the bipolar stimulation parameters used here, finite element modeling indicates that current spread is localized to a discrete region around the bipolar electrode contacts (52). Fourth, despite stimulating 87 sites, we were unable to test whether stimulation of sites within the small nucleus, anterior amygdala area (53, 54), could elicit apnea, because no electrode contacts were located here. Focal stimulation of this small amygdala nucleus will require further study. Moreover, it is beyond the scope of this study to functionally map the whole brain and its causal connections, and thus, it is possible that other sites may also play a role in postictal apnea. Fifth, the human amygdala atlas (55) used to define the amygdala nuclei was based on a study involving healthy individuals and thus normal anatomy. Consequently, there is a possibility of anatomical variations in our epilepsy cohort compared with the atlas. However, no structural abnormalities were detected on MRI in the patients included in this report, justifying the use of this atlas. Sixth, our patient-subject cohort includes a wide range of ages, and the amygdala undergoes functional changes during development (56, 57) that could affect our results here. However, we previously reported that amygdala stimulation–induced apnea was similarly observed in adults and children as young as 3 years old. Notably, we found postictal apnea due to amygdala seizures in both pediatric and adult patients in this study.

More data will be needed to better determine the extent of potential differences in postictal apnea vulnerability between patients. Characterizing such differences may have particular value for quantifying risk for SUDEP due to persistent respiratory suppression. Potential for SUDEP may be especially high in patients with an exaggerated propensity for prolonged postictal apnea. Confirming this risk may require longitudinal studies tracking SUDEP in patients with postictal apnea, which is beyond the scope of the present work. Nevertheless, we are saddened to report that one of the patients in this study, P384, subsequently died of probable SUDEP seven weeks after resection surgery. P384 exhibited the most dramatic post-stimulation apnea phenotype in our study. P384 was seen in our clinic 1 month after surgery and was seizure-free to that point. Three weeks later he was found unresponsive, face down next to his bed with hands clenched. Although surgery entailed resection of the right amygdala, persistent apnea and hypoventilation were elicited by stimulation of the left amygdala, which was not resected. It is plausible that the persistent and prolonged inhibition of breathing from a seizure that spread to the left amygdala may have played a role in his death from probable SUDEP. This possibility is further strengthened by multiple preclinical studies, suggesting that the amygdala is critical for postictal apnea and SUDEP (58–60). Further study will be required to assess whether individuals exhibiting persistent apneas or hypoventilation after amygdala stimulation or seizure may represent a population at greater risk of SUDEP.

## Methods

### Patients.

Twenty patients with medically intractable epilepsy (12 adult and 8 pediatric) were studied while undergoing iEEG during a 2-week monitoring period for seizure focus localization (Table 2). The 8 pediatric patients and 2 of the adults were described in a previous report. Types of epilepsy in this cohort ranged from focal to multifocal epilepsy with unilateral or bilateral seizure foci in the temporal, frontal, and parietal lobes. Patients were implanted with intracranial electrodes (Ad-Tech Medical Instrument Corp. or PMT Corp.) at sites determined by the multidisciplinary epilepsy team at the University of Iowa. All patients had at least 1 amygdala depth electrode. Antiepileptic drugs were discontinued during the monitoring period to promote seizure occurrence and were restarted prior to electrical stimulation mapping. Although spontaneous seizures occurred during the monitoring period, clinical protocols important for patient protection and seizure semiology interfered with respiratory data collection and potential apneic episodes during the postictal period. Therefore, spontaneous seizures were not analyzed for postictal respiratory effects in this report. All experimental protocols were approved by the University of Iowa Institutional Review Board. Informed consent was obtained from all patients over 18 years of age and from the parents or legal guardians of all patients under 18 years of age. Verbal assent was obtained from children 5–9 years of age, and written assent was obtained from all older children. No assent was obtained from the 3-year-old who was tested in the company of her parents who had the option of terminating the experimental protocol. Consent could be rescinded at any time without interfering with the patient’s clinical evaluation. Similarly, children could rescind assent at any point.

### Imaging and electrode localization.

Electrode localization was performed via MR and CT imaging using techniques previously reported (12, 61). See Supplemental Methods for details (supplemental material available online with this article; https://doi.org/10.1172/jci.insight.172423DS1).

### Amygdala nuclei parcellation.

Eight amygdala nuclei were delineated and parcellated using the CIT168 human brain template (55). This was conducted using a high-precision nonlinear volumetric coregistration of preoperative structural T1 and T2 imaging onto the template brain within the MNI coordinate space. See Supplemental Methods for details.

### Electrical stimulation.

Direct electrical stimulation with intracranial electrodes is a well-established method to map brain function (12, 62) and induce seizures to identify epileptogenic foci (63, 64). Adjacent electrode contacts (“sites”) were stimulated with a bipolar biphasic waveform with a 200-microsecond pulse width and frequency of 50 Hz at constant voltage (Grass SD9 stimulator) as described previously (12, 13). Stimulation at bedside was conducted when the patients were awake and in a relaxed state, breathing normally. Patients were monitored continuously by cardiorespiratory telemetry, iEEG, and visually via live video feed in an adjacent room. Patients were resting in bed and were not engaged by clinicians or epilepsy monitoring unit technicians during or after stimulation-evoked seizures and electrical stimulation functional mapping until breathing returned to normal. A stimulus-response curve for each subject was obtained by increasing stimulation voltage beginning at 2.5 V until breathing was affected up to a maximum of 15 V (the typical threshold for motor movement with stimulation of the motor cortex in these patients). iEEG was monitored during voltage escalation for evidence of seizure activity. At sites where seizure activity did not occur, functional mapping of apnea induction was carried out. With increasing voltage during establishment of the stimulus response, apnea (absence of breath; see definition and measurement in *Respiratory measurements* section below) was an all-or-none effect. Apnea occurred when stimulation voltage reached 10–15 V in all patients except P403 and P416, consistent with previous findings (12, 13). Stimulation duration varied based on the proximity of the stimulation site to the patient’s presumed seizure focus (range 5–60 seconds); sites near the seizure focus were stimulated for shorter durations. A minimum rest interval of 1.5–2.5 minutes was inserted between trials to allow for a return to respiratory baseline. In cases of persistent apnea or seizure, trials were halted until the patient returned to baseline iEEG and breathing. No subjects showed signs of or reported any pain or discomfort during stimulation. Patients and their parents/guardians were blinded to the timing of electrical stimulation delivery.

### Intraoperative electrical stimulation.

Intraoperative electrical brain stimulation was conducted just prior to electrode removal in the operating room. Intubation was performed under propofol anesthesia in the presence of succinylcholine, which was subsequently reversed, and anesthesia was maintained with sevoflurane and isoflurane, allowing patients to breathe on their own (spontaneously). No other drugs that might alter breathing were administered (e.g., narcotics or benzodiazepines). This allowed for precise measures of breathing via a connected ventilator. Electrical stimulation experiments were then conducted as described above.

### Intracranial recording.

iEEG data were acquired using the Neurofax EEG-1200 Platform with a JE-120 256-channel amplifier and analyzed using Neuroworkbench software (Nihon Kohden Corp.); all subjects except P352 also had iEEG data recorded to a dedicated research computer using a Neuralynx ATLAS amplifier. Patients were monitored at the University of Iowa Hospitals and Clinics or the University of Iowa Stead Family Children’s Hospital. Experimental protocols did not interfere with collection of clinically relevant data. Stimulation-induced seizure foci, onset, and spread were determined by analysis of iEEG by an adult or pediatric epileptologist as well as the epilepsy neurosurgeon.

### Respiratory measurements.

Central apnea (absence of breathing) was defined as at least 1 missed breath with a flattened airflow trace and verified by absence of chest wall movement and/or video confirmation of the absence of chest wall movement. See Supplemental Methods for details of respiratory monitoring equipment. Trials were categorized as “apnea” when breathing was interrupted for the entire stimulation duration and as “transient apnea” when normal baseline breathing resumed before the end of stimulation. “Persistent apnea(s)” was defined as central apnea(s) lasting in total at least 20 seconds beyond the end of stimulation or seizure. The breathing pattern was considered normal when there was at least 20 seconds of regular baseline-like breathing without any apnea. One breath was defined as a complete inspiratory and expiratory cycle. Oxygen desaturation was defined as <90%. All patients had normal baseline breathing without periods of apnea prior to experimental protocols.

Typically, multiple stimulation trials were conducted at each site. The outcome of each stimulation trial was categorized as persistent apnea, apnea, transient apnea, or no apnea. Each stimulated site was categorized based on the respiratory outcome elicited by the majority of stimulation trials. If stimulation of a site resulted in an equal distribution of respiratory outcomes over all trials at that site, it was categorized as being the more prolonged type of observed respiratory effect (e.g., if stimulation resulted in apnea and transient apnea in an equal number of trials, the site was categorized as apnea).

### Data analysis and experimental design.

Data analyses were conducted using Matlab (Mathworks), GraphPad Prism (GraphPad Software Inc.), and Excel (Microsoft). Digitization of breathing traces from operating room video was performed using the WebPlotDigitizer tool (65). See Supplemental Methods for details of iEEG analysis.

### Machine learning–classifier analysis.

To identify a focal site that induced apnea across patients, we trained a classifier to predict the respiratory effect of stimulation based on the location of the stimulated site (13) (Supplemental Table 1). Spatial clustering of stimulation sites associated with 4 different outcomes — no effect, transient apnea, apnea, and persistent apnea — was performed with a multi-class error-correcting output code classifier (66). See Supplemental Methods for details.

### Electrical stimulation concurrent with fMRI.

Electrical stimulation concurrent with fMRI (es-fMRI) and its safety testing have been described previously (18). Preprocessing of the MRI data was done using the standard fMRIPrep pipeline (67). After preprocessing, statistical analysis was performed at the whole-brain level using general linear modeling as implemented in the SPM12 toolbox (https://www.fil.ion.ucl.ac.uk/spm/software/spm12/) at each voxel. See Supplemental Methods for details of fMRI acquisition, preprocessing, and statistical analyses.

### Statistics.

Statistical and data analyses were conducted using Matlab (Mathworks). Classifiers (Figure 4) were specified and fit using, respectively, the Matlab functions “templateSVM” and “fitcecoc” contained in the Matlab Statistics and Machine Learning Toolbox. Post-stimulation/postictal power changes (power and correlation; Figure 6) were assessed by Wilcoxon’s rank sum test (Matlab function “ranksum”) to test whether the changes were significantly different from zero (at *P* value of 0.01). All fMRI data analyses were performed using SPM toolbox. Difference in brain activation between baseline and stimulation period was assessed using *t*-contrasts thresholded at *P* = 0.001.

### Study approval.

Experiments reported in this study were reviewed and approved by the University of Iowa Institutional Review Board. Written informed consent was obtained from all patients over 18 years of age and from the parents or legal guardians of all patients under 18 years of age. Verbal assent was obtained from children 5–9 years of age, and written assent was obtained from children aged 10–17.

### Data availability.

Individual data points for Figure 5 and values contributing to averages reported in Figure 6 are available in the Supporting Data Values file. Raw data reported in this article will be made available upon reasonable request by qualified individuals upon completion of a data transfer and use agreement, owing to the sensitive nature of intracranial recordings obtained in a clinical setting. Requests may be made to the corresponding author.

## Author contributions

GISH, AER, BJD, and JAW designed the study. GISH, AER, BJD, YN, HK, MAH, and RNM performed study procedures. GISH, AER, CKK, SK, MRM, BJD, and HO analyzed data. GISH, AER, BJD, CKK, SK, MRM, RKS, BKG, MAC, KTSP, KS, MS, PWD, ACC, JAW, and GBR interpreted data. GISH, AER, BJD, and JAW wrote the first draft of the manuscript. All authors contributed to revisions of the manuscript. Co–first author order was determined alphabetically by last name.

## Supplementary Material

Supplemental data

Supporting data values

## Figures and Tables

**Table 2:**
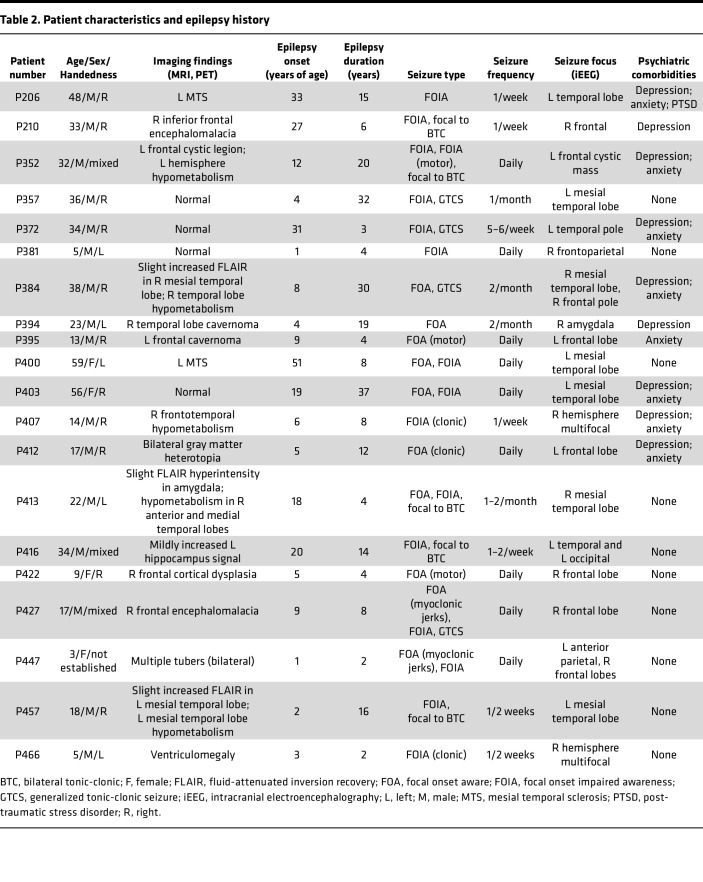
Patient characteristics and epilepsy history

**Table 1:**
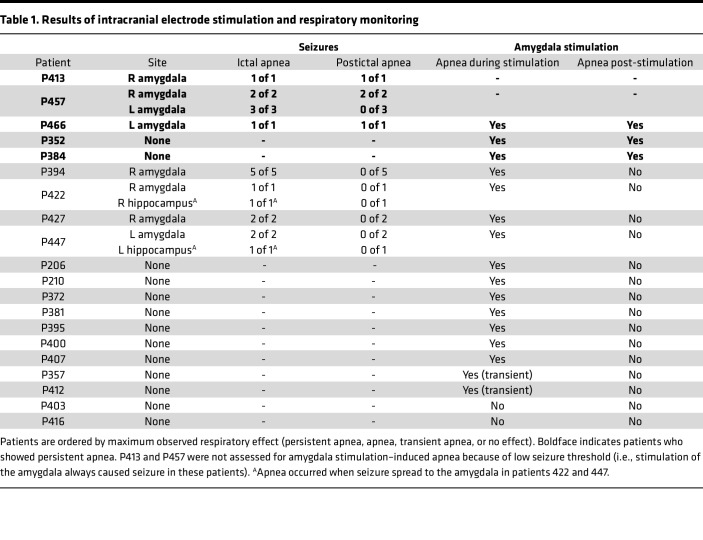
Results of intracranial electrode stimulation and respiratory monitoring

## Version 1 10/03/2023

In-Press Preview

## Version 2 11/22/2023

Electronic publication
