# Supplementary material for: Failure to breathe persists without air hunger or alarm following amygdala seizures
Source: JCI Insight. 2023 Nov 22;8(22):e172423. doi: 10.1172/jci.insight.172423 (PMC10721319; doi:10.1172/jci.insight.172423)
Supplement: Supplemental data [file jciinsight-8-172423-s217.pdf]

Supplement: Failure to breathe persists without air hunger or alarm following amygdala seizures

Gail I. S. Harmata[1,2,3,4,5,6] †, Ariane E. Rhone[1,2]†, Christopher K. Kovach[1,2], Sukhbinder Kumar[1], Md Rakibul Mowla[1], Rup K. Sainju[7], Yasunori Nagahama[1]‡, Hiroyuki Oya[1,2], Brian K. Gehlbach[8], Michael A. Ciliberto[9], Rashmi N. Mueller[1,10], Hiroto Kawasaki[1], Kyle T.S. Pattinson[11], Kristina Simonyan[12], Paul W. Davenport[13], Matthew A. Howard III[1,2,3], Mitchell Steinschneider[14], Aubrey C. Chan[6,8], George B. Richerson[2,3,4,7,15,16], John A. Wemmie[1,2,3,4,6,8,14,16], Brian J. Dlouhy[1,2,3]*

Affiliations:

[1]Department of Neurosurgery, University of Iowa; Iowa City, IA 52242, USA. [2]Iowa Neuroscience Institute, University of Iowa; Iowa City, IA 52242, USA. [3]Pappajohn Biomedical Institute, University of Iowa; Iowa City, IA 52242, USA. [4]Interdisciplinary Graduate Program in Neuroscience, University of Iowa; Iowa City, IA 52242, USA. [5]Pharmacological Sciences Training Program, University of Iowa; Iowa City, IA 52242, USA. [6]Department of Psychiatry, University of Iowa; Iowa City, IA 52242, USA.

[7]Department of Neurology, University of Iowa; Iowa City, IA 52242, USA. [8]Department of Internal Medicine, University of Iowa; Iowa City, IA 52242, USA. [9]Department of Pediatrics, University of Iowa; Iowa City, IA 52242, USA. [10]Department of Anesthesia, University of Iowa; Iowa City, IA 52242, USA.

[11]Nuffield Department of Clinical Neurosciences, University of Oxford, John Radcliffe Hospital; Oxford, OX3 9DU, UK. [12]Department of Otolaryngology – Head and Neck Surgery, Massachusetts Eye and Ear and Harvard Medical School; Boston, MA 02114, USA. [13]Department of Physiological Sciences, University of Florida; Gainesville, FL 32610, USA. [14]Department of Neurology, Albert Einstein College of Medicine; Bronx, NY 10461, USA. [15]Department of Molecular Physiology and Biophysics, University of Iowa; Iowa City, IA 52242, USA. [16]Department of Veterans Affairs Medical Center; Iowa City, IA 52246, USA.

*Corresponding author:

Brian J. Dlouhy, Department of Neurosurgery, University of Iowa Carver College of Medicine, University of Iowa Stead Family Children's Hospital, 200 Hawkins Drive, Iowa City, Iowa 52242, USA. Phone: 319.356.2542; Email: brian-dlouhy@uiowa.edu.

† Co-first authors

‡ Current affiliation: Department of Neurosurgery, Robert Wood Johnson Medical School, Rutgers University; New Brunswick, NJ 08901, USA.

Supplemental Contents:

Supplemental Methods

Supplemental Table 1: MNI Coordinates of Stimulated Sites

Supplemental Methods

Imaging and electrode Localization

For each patient, whole-brain high-resolution T1-weighted structural MR scans (resolution and slice thickness ≤1.0 mm) were obtained before and after electrode implantation. Post-implantation CT and 3T MR structural scans were linearly coregistered to pre-implantation MR scans using the FLIRT module of FSL [1]. These images were corrected manually for displacement and deformation from surgery using nonlinear thin-plate spline warping using manually selected control points. Finally, FreeSurfer was used to identify the cortical surface and white/gray boundaries within pre-implantation T1 images, which were corrected manually based on visual inspection where necessary.

Amygdala nuclei parcellation

Eight amygdala nuclei were delineated according to the probabilistic atlas of Tyszka and Pauli [2], and the structural relationships between electrode contacts and nuclei were determined from the projected contact locations within the preoperative T1 image space as described previously [3]. The nuclei included: lateral, basolateral, basomedial, central, and cortical/medial as well as amygdala transition areas, amygdalostriatal transition area, and anterior amygdala area. This atlas used to delineate amygdala subregions [2] provides more accurate external boundary definition than what has previously been estimated with histology-based atlases in humans or other nuclei parcellations from other sources. This atlas has been used in numerous publications validating its use across human studies and has been cited more than 100 times since its publication in 2016.

Respiratory Monitoring Equipment

Oral and nasal airflow at bedside were measured with a BiNAPS nasal pressure transducer and a ThermiSense oral/nasal thermistor (SunMed, Grand Rapids, MI). Chest and abdominal excursions were recorded using plethysmography belts (ProTech zRIP). Arterial oxygen saturation (SpO_2) was recorded using a BluPro SpO_2 monitor. In the operating room, respiratory monitoring was evaluated using an Aisys Carestation with B850 monitor (GE/Datex-Ohmeda, Inc.), which allowed for real-time anesthesia and respiratory evaluation of respiratory rate, tidal volume, minute ventilation, and $etCO_2$. This was video recorded for later evaluation.

Analysis of iEEG power

Time-frequency analysis of iEEG data was performed by first filtering the data into narrow frequency bands with center frequencies ranging from 1 to 150 Hz in steps of 1 Hz. Filtering was done by convolving the data with Gaussian kernel with width-at-half-maximum set to 5. Signal power for each frequency band was calculated by taking the square of the amplitude envelope computed using the Hilbert transform. To test if there was a significant change in iEEG power after stimulation, the frequency range was divided into 6 bands: delta (1-4Hz); theta (4-8 Hz); alpha (8-12 Hz); beta (12-30Hz); low gamma (30-80Hz) and high gamma (80-150Hz) and change in average (over time) power for each of the frequency bands was calculated with respect to a baseline of 30 s before the stimulation onset. The power changes were then subjected to statistical analysis using Wilcoxon rank sum test (Matlab function: ranksum) to test if the changes were significantly different from zero. To test if the breathing pattern after stimulation onset correlated with fluctuations in power in any of the frequency bands, the breathing trace was first low pass filtered to 1 Hz followed by calculation of the amplitude envelope using the Hilbert transform. The correlation coefficient between the amplitude envelope of breathing and the amplitude envelope of each of the frequency bands was calculated both during the post-stimulation period and

during a baseline period of 30 s before stimulation onset. Changes in correlation coefficient with respect to baseline were then subjected to statistical analysis using Wilcoxon rank sum test as above.

Machine Learning - Classifier Analysis

Classifier analysis (Figure 4, main text) was performed multi-class error correcting output code (ECOC) classifier [4]. ECOC classifiers adapt binary classifiers to multiway classification by fitting multiple redundant binary classifiers across binary subgroupings of the outcome categories and treating the combined output of all classifiers as an error correcting code, where each classifier contributes one bit of information. The predicted class for a given input is obtained according to the minimum of a distance metric between the combined binary classifier outputs and binary values encoding the respective classes. Binary classifiers in the present application were 4 kernel support vector machines (SVM), each trained to distinguish one outcome class from all others with the midpoint between cathode and anode coordinates in MNI space as the predictor, using a 6.7 mm FWHM Gaussian spatial kernel. Posterior class probabilities were estimated with 10-fold cross validation, and the distance metric used in class prediction was quadratic loss with respect to the estimated posterior probabilities across binary classifiers. Classifiers were specified and fit using, respectively, the Matlab functions, "templateSVM", and "fitcecoc," contained in the Matlab Statistics and Machine Learning Toolbox.

Electrical stimulation concurrent with fMRI (es-fMRI)

fMRI data were acquired during stimulation on a 3T Siemens Skyra scanner with the following parameters: TR = 3 s; TE = 30 ms; voxel size =3 cubic mm isotropic; delay between successive volume acquisitions = 100 ms. Electrical stimulation was done during the delay between scans. Bipolar, biphasic electrical stimulation was conducted using an isolated biphasic stimulus generator (A-M Systems, Sequim, WA, USA). A pre-electrode implantation T1-weighted anatomical MRI scan was acquired with the following parameters: 32 channel head coil, TR = 8.588 ms; TE =3.376 ms; Flip angle =12 degrees; voxel size =1.0 x 1.0 x 0.8 mm. Further details of the data acquisition can be found in Thompson et al.[5].

Pre-processing of the fMRI data was done using the standard fMRIPrep pipeline [6]. After correction for intensity non-uniformity, the anatomical T1w scan was skull-stripped and segmented into gray matter, white matter, and cerebrospinal fluid. The anatomical scan was then normalized to MNI space through nonlinear registration with a T1w template. The fMRI data were pre-processed using the following steps: i) generation of a reference volume from the data collected in one session; ii) field correction using fMRIPrep's firedamp-less approach; (iii) head-motion correction and estimation of motion parameters; (iv) co-registration of the BOLD reference with the anatomical scan; (v) slice-time correction; (vi) normalization to MNI space; and (vii) smoothing with a Gaussian kernel of 8mm FWHM (full-width-at-half-maximum). More details of the pre-processing can be found in Thompson et al.[5].

Statistical analysis of es-fMRI

Onset times of electrical stimulation pulses were entered as events and then convolved with the canonical hemodynamic response function. In addition, the design matrix consisted of the following 22 nuisance regressors: (i) 6 motion parameters; (ii) 6 first derivative of the motion parameters; (iii) 8 RETROICOR regressors (up to 4th harmonic) of the respiration trace [7]; (iv) 1 respiratory volume time (RVT) regressor modelling change in respiration rate or depth over time [8]; and (v) 1 regressor for removing linear trend in the data. A high-pass filter of 1/128 Hz was applied to remove low frequency fluctuations in the BOLD signal. After the GLM model was estimated, two contrasts (Stimulation ON > Stimulation OFF and Stimulation OFF > Stimulation ON) were estimated. Clusters of activations were determined using a peak

voxel threshold of p =0.001 with (cluster) extent threshold of 30 voxels. BOLD time series from the insula and brain stem clusters (Figure 7E, main text) were computed using principal component analysis of voxels in the cluster and retaining the first principal component ('eigenvariate' function in the SPM toolbox) after adjusting for nuisance regressors. The t-values for the brain stem and insula clusters (Figure 7I, main text) corresponded to the maxima of cluster in the first-level t-maps.

1. Jenkinson, M. and S. Smith, *A global optimisation method for robust affine registration of brain images.* Med Image Anal, 2001. **5**(2): p. 143-56.
2. Tyszka, J.M. and W.M. Pauli, *In vivo delineation of subdivisions of the human amygdaloid complex in a high-resolution group template.* Hum Brain Mapp, 2016. **37**(11): p. 3979-3998.
3. Rhone, A.E., et al., *A human amygdala site that inhibits respiration and elicits apnea in pediatric epilepsy.* JCI Insight, 2020. **5**: p. e134852.
4. Dietterich, T.G. and G. Bakiri, *Solving Muticlass Learning Problems via Error-Correcting Output Codes.* J Artificial Intelligence Research, 1995. **2**: p. 263-286.
5. Thompson, W.H., et al., *A data resource from concurrent intracranial stimulation and functional MRI of the human brain.* Scientific Data, 2020. **7**(1): p. 258.
6. Esteban, O., et al., *fMRIPrep: a robust preprocessing pipeline for functional MRI.* Nat Methods, 2019. **16**(1): p. 111-116.
7. Glover, G.H., T.Q. Li, and D. Ress, *Image-based method for retrospective correction of physiological motion effects in fMRI: RETROICOR.* Magn Reson Med., 2000. **44**(1): p. 162-7.
8. Birn, R.M., et al., *The respiration response function: the temporal dynamics of fMRI signal fluctuations related to changes in respiration.* NeuroImage, 2008. **40**(2): p. 644-654.

Supplemental Table 1: MNI Coordinates of Stimulated Sites

Patient	AgeGroup	Effect	X1	Y1	Z1	X2	Y2	Z2	SiteX*	SiteY	SiteZ	Site Nucleus/ROI
206	adult	Apnea	23.77	-3.07	-22.26	33.33	-3.31	-23.86	28.55	-3.19	-23.06	BL/LA
210	adult	Apnea	-12.07	-5.45	-17.81	-23.85	-4.92	-17.38	17.96	-5.185	-17.595	ATA/CMN/BM/BL
210	adult	Apnea	24.88	-5.23	-25.08	38.99	-2.36	-26.41	31.935	-3.795	-25.745	LA
210	adult	No effect	38.99	-2.36	-26.41	43.57	-1.72	-22.98	41.28	-2.04	-24.695	WM
352	adult	No effect	-16.88	-1.81	-26.09	-27.34	0.05	-30.24	22.11	-0.88	-28.165	BL/LA
352	adult	No effect	-27.34	0.05	-30.24	-36.43	1.48	-29.27	31.885	0.765	-29.755	WM
352	adult	Persistent Apnea	16.14	-3.21	-21.66	27.28	-3.93	-21.23	21.71	-3.57	-21.445	BL/LA
352	adult	No effect	27.28	-3.93	-21.23	37.04	-6	-20.01	32.16	-4.965	-20.62	LA/WM
357	adult	Transient Apnea	-22.75	-5.2	-27.25	-27.93	-4.77	-26.49	25.34	-4.985	-26.87	LA
357	adult	No effect	-27.93	-4.77	-26.49	-32.19	-4.85	-26.85	30.06	-4.81	-26.67	LA/WM
372	adult	No effect	-28.91	-34.9	-12.68	-31.19	-31.07	-12.04	30.05	-32.985	-12.36	Hipp
372	adult	No effect	-14.96	-10.43	-22.01	-19.83	-8.48	-21.78	17.395	-9.455	-21.895	Hipp/BL
372	adult	Apnea	-19.83	-8.48	-21.78	-24.07	-7.44	-22.06	21.95	-7.96	-21.92	BL
372	adult	No effect	-24.07	-7.44	-22.06	-28.62	-6.16	-21.96	26.345	-6.8	-22.01	BL/LA
372	adult	No effect	-28.62	-6.16	-21.96	-33.54	-5	-21.67	31.08	-5.58	-21.815	LA/WM
372	adult	No effect	-33.54	-5	-21.67	-38.88	-3.3	-20.28	36.21	-4.15	-20.975	WM
381	pediatric	Apnea	-16.03	-0.95	-22.28	-19.46	-0.37	-22.41	17.745	-0.66	-22.345	ATA/BL/BM
381	pediatric	Transient Apnea	-19.46	-0.37	-22.41	-23.49	-0.2	-22.63	21.475	-0.285	-22.52	BL
381	pediatric	No effect	-23.49	-0.2	-22.63	-27.61	-0.16	-22.97	25.55	-0.18	-22.8	LA
381	pediatric	No effect	-27.61	-0.16	-22.97	-32.42	0.07	-23.21	30.015	-0.045	-23.09	LA
381	pediatric	No effect	-32.42	0.07	-23.21	-36.29	-1.02	-23.29	34.355	-0.475	-23.25	WM
384	adult	Persistent Apnea	-18.12	-1.08	-21.2	-28.4	1.41	-20.71	23.26	0.165	-20.955	BM/BL/LA
384	adult	No effect	-28.4	1.41	-20.71	-36.92	4.1	-19.2	32.66	2.755	-19.955	LA/WM
384	adult	No effect	10.63	5.77	-17.7	21.13	7.66	-17.42	15.88	6.715	-17.56	orbitofrontal
384	adult	No effect	26.77	-29.3	-14.72	33.82	-20.61	-14.24	30.295	-24.955	-14.48	Hipp
384	adult	No effect	33.82	-20.61	-14.24	40.06	-11.81	-15.4	36.94	-16.21	-14.82	Hipp/WM
384	adult	Transient Apnea	23.18	-10.44	-11.2	32.88	-13.29	-8.52	28.03	-11.865	-9.86	CMN/WM
384	adult	Transient Apnea	19.58	-4.32	-22.89	24.9	-2.59	-23.63	22.24	-3.455	-23.26	BL/LA
384	adult	No effect	24.9	-2.59	-23.63	30.3	-0.81	-23.34	27.6	-1.7	-23.485	LA
384	adult	No effect	30.3	-0.81	-23.34	35.22	0.22	-22.98	32.76	-0.295	-23.16	LA/WM
384	adult	No effect	35.22	0.22	-22.98	40.97	1.1	-21.12	38.095	0.66	-22.05	WM
394	adult	No effect	6.66	15.5	-20.04	17.69	12.18	-19.54	12.175	13.84	-19.79	orbitofrontal
394	adult	No effect	51.36	-17.71	-5.18	56.16	-15.19	-7.76	53.76	-16.45	-6.47	WM
394	adult	Apnea	18.19	-8.31	-23.71	20.89	-8.45	-23.83	19.54	-8.38	-23.77	BL
394	adult	Apnea	23.6	-8.63	-24.1	26.26	-8.66	-24.23	24.93	-8.645	-24.165	BL/LA
394	adult	Apnea	26.26	-8.66	-24.23	28.75	-8.53	-24.17	27.505	-8.595	-24.2	LA
394	adult	No effect	28.75	-8.53	-24.17	31.08	-8.31	-24.14	29.915	-8.42	-24.155	LA
395	pediatric	No effect	-22.25	-32.46	-10.59	-27.32	-31.01	-10.14	24.785	-31.735	-10.365	Hipp
395	pediatric	No effect	-27.32	-31.01	-10.14	-32.91	-29.9	-9.33	30.115	-30.455	-9.735	Hipp
395	pediatric	No effect	-10.27	-1.81	-22.19	-12.29	-2.13	-21.24	11.28	-1.97	-21.715	ATA/CMN

395	pediatric	Apnea	-12.29	-2.13	-21.24	-14.56	-2.41	-20.51	13.425	-2.27	-20.875	ATA/CMN/BM
395	pediatric	Apnea	-14.56	-2.41	-20.51	-16.69	-2.84	-19.78	15.625	-2.625	-20.145	BM
395	pediatric	Apnea	-16.69	-2.84	-19.78	-19.52	-3.19	-19.65	18.105	-3.015	-19.715	BM/BL
395	pediatric	Apnea	-19.52	-3.19	-19.65	-22.42	-3.52	-19.95	20.97	-3.355	-19.8	BL/LA
395	pediatric	Transient Apnea	-22.42	-3.52	-19.95	-25.08	-4.06	-20.26	23.75	-3.79	-20.105	LA
395	pediatric	Transient Apnea	-25.08	-4.06	-20.26	-27.49	-4.43	-20.41	26.285	-4.245	-20.335	LA
400	adult	No effect	-29.74	-32.67	-12.8	-37.21	-24.81	-12.7	33.475	-28.74	-12.75	Hipp
400	adult	Apnea	-20.12	-10.05	-23.81	-28.86	-5.75	-24.57	24.49	-7.9	-24.19	LA/BL/Hipp
403	adult	No effect	-33.81	8.41	-13.92	-44.17	8.88	-12.84	38.99	8.645	-13.38	orbitofrontal
403	adult	No effect	-28.07	-7.83	-25.06	-30.05	-7.23	-24.57	29.06	-7.53	-24.815	Hipp
403	adult	No effect	-30.05	-7.23	-24.57	-31.84	-6.81	-24.3	30.945	-7.02	-24.435	WM
403	adult	No effect	-31.84	-6.81	-24.3	-33.65	-6.43	-24.31	32.745	-6.62	-24.305	WM
407	pediatric	Apnea	16.28	-6.57	-21.27	18.73	-6.58	-21.15	17.505	-6.575	-21.21	BL
412	pediatric	Transient Apnea	-23.13	0.08	-23.05	-25.7	-0.41	-22.57	24.415	-0.165	-22.81	LA
412	pediatric	No effect	-37.79	-1.55	-21.3	-40.38	-1.69	-21.12	39.085	-1.62	-21.21	WM
413	adult	Persistent Apnea	20.04	-2.33	-27.29	21.73	-1.6	-27.66	20.885	-1.965	-27.475	BL
416	adult	No effect	-23.17	-4.54	-13.2	-25.34	-5.51	-13.02	24.255	-5.025	-13.11	BL/BM/CEN/ASTA
422	pediatric	Apnea	15.12	-3.89	-24.38	18.15	-4.53	-23.87	16.635	-4.21	-24.125	BL
422	pediatric	Apnea	18.15	-4.53	-23.87	21.48	-4.98	-23.43	19.815	-4.755	-23.65	BL
422	pediatric	Apnea	21.48	-4.98	-23.43	24.6	-5.1	-22.99	23.04	-5.04	-23.21	BL
422	pediatric	Transient Apnea	24.6	-5.1	-22.99	27.41	-5.07	-22.46	26.005	-5.085	-22.725	BL/LA
422	pediatric	No effect	25.43	-18.21	-18.26	30.78	-18.77	-17.83	28.105	-18.49	-18.045	Hipp
427	pediatric	Apnea	-14.66	-6.42	-23.38	-16.93	-6.78	-23.23	15.795	-6.6	-23.305	BL
427	pediatric	Apnea	-16.93	-6.78	-23.23	-19.22	-7.17	-23.04	18.075	-6.975	-23.135	BL
427	pediatric	Transient Apnea	-19.22	-7.17	-23.04	-21.51	-7.51	-22.84	20.365	-7.34	-22.94	BL
427	pediatric	No effect	-14.64	-13.65	-22.29	-20.68	-14.38	-20.99	17.66	-14.015	-21.64	Hipp
427	pediatric	No effect	-20.68	-14.38	-20.99	-26.21	-15.21	-20.12	23.445	-14.795	-20.555	Hipp
427	pediatric	Apnea	14.75	-8.42	-19.09	17.15	-9.04	-18.52	15.95	-8.73	-18.805	BL/ATA
447	pediatric	No effect	-13.12	-11.26	-18.16	-15.86	-10.92	-17.51	14.49	-11.09	-17.835	ATA
447	pediatric	Transient Apnea	-15.86	-10.92	-17.51	-18.7	-10.51	-17.02	17.28	-10.715	-17.265	ATA/BL/CMN
447	pediatric	Apnea	-18.7	-10.51	-17.02	-21.66	-9.73	-16.7	20.18	-10.12	-16.86	CMN/BL/BM
447	pediatric	No effect	-21.66	-9.73	-16.7	-24.66	-8.64	-16.52	23.16	-9.185	-16.61	BL/BM
447	pediatric	No effect	-24.66	-8.64	-16.52	-27.56	-7.67	-16.52	26.11	-8.155	-16.52	BL
447	pediatric	No effect	-27.56	-7.67	-16.52	-30.29	-7.06	-16.78	28.925	-7.365	-16.65	BL/lateral to amygdala
447	pediatric	No effect	-24.03	-30.72	-8.64	-29.88	-29.2	-9.5	26.955	-29.96	-9.07	Hipp
447	pediatric	Apnea	14.34	0.41	-17.71	17.18	0.67	-17.22	15.76	0.54	-17.465	CMN
447	pediatric	Apnea	17.18	0.67	-17.22	19.93	1.05	-16.92	18.555	0.86	-17.07	CMN
447	pediatric	No effect	19.93	1.05	-16.92	22.4	1.6	-16.82	21.165	1.325	-16.87	anterior to amygdala
447	pediatric	No effect	22.4	1.6	-16.82	24.67	2.29	-16.95	23.535	1.945	-16.885	anterior to amygdala
447	pediatric	No effect	24.67	2.29	-16.95	27.12	2.95	-17.47	25.895	2.62	-17.21	anterior to amygdala
447	pediatric	No effect	27.12	2.95	-17.47	29.83	3.64	-18.31	28.475	3.295	-17.89	anterior to amygdala
457	adult	Apnea	-13.45	-7.88	-18.99	-15.69	-7.78	-18.65	14.57	-7.83	-18.82	ATA/BL

457	adult	Persistent Apnea	15.59	-1.2	-20.95	18.32	-1.29	-20.75	16.955	-1.245	-20.85	ATA/BL
466	pediatric	No effect	23.74	-25.97	-15.29	28.06	-24.08	-14.75	25.9	-25.025	-15.02	Hipp
466	pediatric	Persistent Apnea	-16.31	-2.81	-21.26	-18.47	-2.88	-21.68	17.39	-2.845	-21.47	CMN/BM
466	pediatric	No effect	-29.16	-2.21	-23.2	-31.42	-1.44	-23.34	30.29	-1.825	-23.27	LA
466	pediatric	Apnea	19.87	-5.47	-21.55	22.38	-5.45	-21.77	21.125	-5.46	-21.66	BL

MNI coordinates, amygdala nucleus assignment, and respiratory outcome for each stimulated site. Sites involved in persistent apnea are shaded gray for emphasis.*For mapping to template brain, all coordinates were plotted on the Right hemisphere (i.e. all SiteX values are positive). La, lateral nucleus; BL, basolateral nucleus; BM, basomedial nucleus; CEN, central nucleus; CMN, cortical and medial nuclei; ATA, amygdala transition areas; ASTA, amygdalostriatal transition area; AAA, anterior amygdala area; Hipp, hippocampus; WM, white matter.
